# Structural mechanism for replication origin binding and remodeling by a metazoan origin recognition complex and its co-loader Cdc6

**DOI:** 10.1038/s41467-020-18067-7

**Published:** 2020-08-26

**Authors:** Jan Marten Schmidt, Franziska Bleichert

**Affiliations:** 1 Friedrich Miescher Institute for Biomedical Research, Maulbeerstrasse 66, Basel, 4058 Switzerland; 2 University of Basel, Petersplatz 1, Basel, 4051 Switzerland; 3 Department of Molecular Biophysics and Biochemistry, Yale University, 260 Whitney Avenue, New Haven, CT 06520 USA

**Keywords:** DNA, Enzyme mechanisms, Replisome, Cryoelectron microscopy

## Abstract

Eukaryotic DNA replication initiation relies on the origin recognition complex (ORC), a DNA-binding ATPase that loads the Mcm2–7 replicative helicase onto replication origins. Here, we report cryo-electron microscopy (cryo-EM) structures of DNA-bound *Drosophila* ORC with and without the co-loader Cdc6. These structures reveal that Orc1 and Orc4 constitute the primary DNA binding site in the ORC ring and cooperate with the winged-helix domains to stabilize DNA bending. A loop region near the catalytic Walker B motif of Orc1 directly contacts DNA, allosterically coupling DNA binding to ORC’s ATPase site. Correlating structural and biochemical data show that DNA sequence modulates DNA binding and remodeling by ORC, and that DNA bending promotes Mcm2–7 loading in vitro. Together, these findings explain the distinct DNA sequence-dependencies of metazoan and *S. cerevisiae* initiators in origin recognition and support a model in which DNA geometry and bendability contribute to Mcm2–7 loading site selection in metazoans.

## Abstract (web summary)

The origin recognition complex (ORC) is essential for loading the Mcm2–7 replicative helicase onto DNA during DNA replication initiation. Here, the authors describe several cryo-electron microscopy structures of *Drosophila* ORC bound to DNA and its cofactor Cdc6 and also report an in vitro reconstitution system for *Drosophila* Mcm2–7 loading, revealing unexpected features of ORC’s DNA binding and remodeling mechanism during Mcm2–7 loading.

## Introduction

The loading of ring-shaped, replicative helicases onto DNA constitutes a key event during the initiation of chromosomal DNA replication. Across all domains of life, replicative helicases are recruited and deposited onto DNA at or near specialized chromosomal regions, termed origins, by the action of dedicated initiator and loader proteins^1,2^. A critical task of initiators during this process is to recognize helicase loading sites and to remodel origins prior to helicase recruitment. The structural choreography that ultimately leads to helicase loading is only beginning to be understood in the different initiation systems.

Cellular replication initiators share a modular domain organization that optimally supports origin recognition and remodeling^3,4^. A helix-turn-helix (HTH) fold serves as the primary origin DNA-binding and origin-targeting region in bacterial and archaeal initiators^5–7^. This HTH domain is appended to an ATP hydrolase associated with various cellular activities (AAA+) fold, a domain prevalent in a large group of proteins involved in diverse biological processes beyond DNA replication^8,9^. A common theme among members of this superfamily is that they act as molecular motors or switches to remodel client molecules bound in the central pore of ring-shaped or helical AAA+ assemblies in response to ATP binding and hydrolysis^10,11^. In initiators, the AAA+ regions also engage DNA, and these interactions directly remodel origin DNA in the prokaryotic proteins^6,7,12^. However, the extent to which the initiator HTH and AAA+ modules contribute to DNA binding and remodeling across the eukaryotic domain is not fully understood. How origin DNA binding is sensed by initiators and coupled to the ATPase sites in the AAA+ oligomer and how this mechanism relates to other AAA+ systems is also unclear.

In many eukaryotes, including fungi, plants, and metazoa, the initiator is a heterohexameric assembly, termed origin recognition complex (ORC), that acts in concert with the co-loaders Cdc6 and Cdt1 to deposit the hexameric Mcm2–7 helicase core onto DNA^1,13^. ORC is composed of five evolutionary related AAA+ proteins (Orc1–5) and a sixth subunit (Orc6) of distinct origin^14,15^. In Orc1–5, the AAA+ modules are augmented by a C-terminal HTH domain of the winged-helix (WH) type and co-assemble into a pentameric ring that can exist in both open (active) or closed (autoinhibited) configurations^16–18^. Conversely, Orc6 is recruited to the periphery of the ORC ring through its interaction with Orc3^16,19^. Structural studies have revealed that the initiator binds DNA in the center of the ORC ring, bending DNA to prepare origins for Mcm2–7 recruitment and loading^18,20,21^. A recent cryo-EM structure of *S. cerevisiae* ORC has further detailed how the AAA+ and WH domains, in conjunction with several basic patch regions, recognize specific autonomous replication sequence (ARS) DNA elements at budding yeast origins^20^. Notably, sequence-specific DNA binding as exemplified by *S. cerevisiae* ORC is a highly specialized mode of origin recognition outside prokaryotic lineages. Consensus sequences are not present at origins in more distantly related fungi or in multicellular eukaryotes; instead, contextual DNA sequence and chromatin cues appear to target ORC to preferred chromosomal regions in these systems^22–31^. Yet, the physical basis for how ORC engages DNA in the absence of specific consensus DNA motifs remains poorly defined. Whether DNA remodeling is required for Mcm2–7 loading is likewise uncertain. Lastly, whether and how the recruitment of co-loaders such as Cdc6 (a AAA+ ATPase itself) elicits conformational changes in ORC and/or DNA prior to Mcm2–7 recruitment is also not understood.

To resolve these outstanding questions, we determined cryo-EM structures of DNA-bound *Drosophila* ORC (*Dm*ORC) assemblies in the presence and absence of the co-loader Cdc6. These structures, in conjunction with biochemical assays examining DNA binding and ATP hydrolysis activities of mutant *Dm*ORC assemblies, reveal unexpected differences to the *S. cerevisiae* ORC·DNA cryo-EM structure^20^ and indicate that the roles of the AAA+ and WH domains in DNA binding have diverged not only among the eukaryotic Orc1–5 subunits, but also between metazoan and related archaeal initiators. We further reconstitute Mcm2–7 loading in vitro using recombinant *Drosophila* proteins and find that DNA bending facilitates Mcm2–7 loading. Together our results suggest mechanistic models for the coupling of DNA binding to the ATPase sites within ORC and ORC·Cdc6, and for the role of DNA sequence context both in targeting of metazoan ORC to origins and in Mcm2–7 loading.

## Results

### Structure of the *Dm*ORC·DNA·Cdc6 complex

**Fig. 1:**
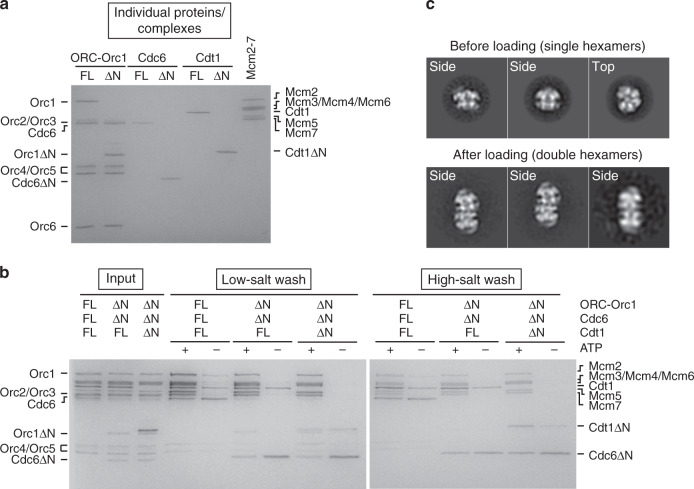
Mcm2–7 loading does not require intrinsically disordered N-terminal regions (IDRs) of loading factors. **a** SDS-PAGE gel of purified *Drosophila* loading factors, either full-length proteins or constructs lacking IDRs (ΔN). **b** *Dm*Mcm2–7 is recruited (low-salt wash) and loaded (high-salt wash) in an ATP-dependent manner by full-length and IDR-less *Dm*Orc1, *Dm*Cdc6, and *Dm*Cdt1 with similar efficiency. Note that FL-Cdt1, Cdt1ΔN, Cdc6, Cdc6ΔN, and to some extent FL-Orc1 associate with beads nonspecifically. **c** Loaded *Dm*Mcm2–7 are double-hexamers. *Dm*Mcm2–7 before and after loading was analyzed by negative-stain EM. 2D class averages show Mcm2–7 single-hexamers before loading but double-hexamers after loading. Source data are provided as a Source data file.

Previous attempts to determine 3D structures of *Drosophila* ORC·DNA or ORC·DNA·Cdc6 by cryo-EM were unsuccessful because the particles adopt a strong preferred orientation on continuous carbon support EM grids and aggregate at the higher protein concentrations required for imaging without carbon layer^18^. Through testing of various deletion constructs of ORC and Cdc6, we found that removal of the N-terminal regions of Orc1 (Orc1ΔN) and Cdc6 (Cdc6ΔN) improves solubility in the presence of DNA and monodispersity of the sample after freezing. These regions precede the AAA+ domains, are predicted to be mostly disordered, and have been reported to drive liquid-liquid phase separation^32^. Deletion of Orc1 and Cdc6 N-termini did not substantially weaken *Dm*ORC’s ATP-dependent affinity for DNA^18^, nor did it impair the ability of *Dm*ORC to hydrolyze ATP and of *Dm*ORC and *Dm*Cdc6 to co-associate in the presence of ATP and DNA (Supplementary Fig. 1). We also established an in vitro helicase loading assay that supported the ATP-dependent, salt-resistant association of *Drosophila* Mcm2–7 with DNA and Mcm2–7 double hexamer formation in the presence of full-length loading factors (Fig. 1). Importantly, *Dm*Orc1, *Dm*Cdc6, but also *Dm*Cdt1 proteins lacking N-terminal, intrinsically disordered regions (IDRs) loaded Mcm2–7 onto DNA to similar extents as full-length proteins (Fig. 1a, b). Together, these findings indicate that the truncated *Dm*ORC and *Dm*Cdc6 constructs are functional and that the IDRs in Orc1, Cdc6, and Cdt1 are not essential for Mcm2–7 loading in vitro.

**Fig. 2:**
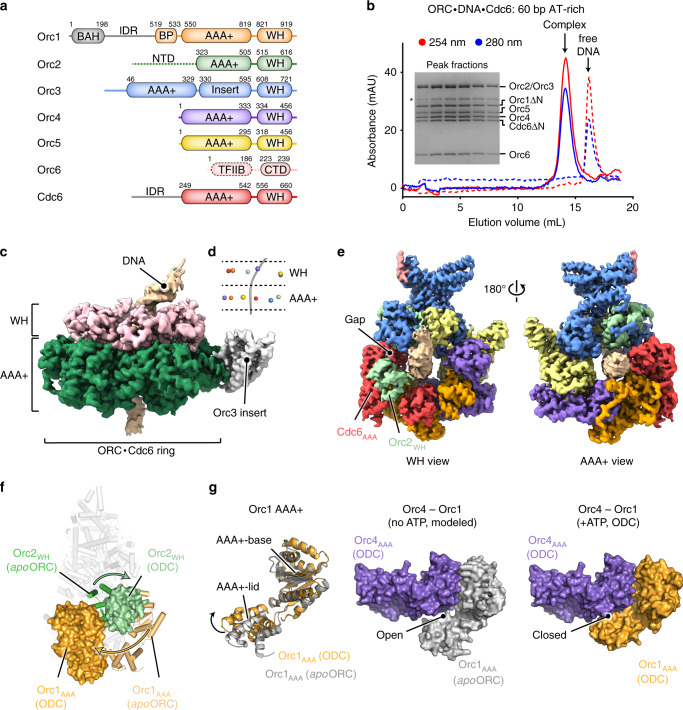
Structure of a licensing-competent *Dm*ORC·DNA·Cdc6 complex. **a** Domain architecture of *Dm*ORC subunits and Cdc6. Dashed lines demarcate regions that are flexible and structurally not resolved. Gray regions in Orc1 and Cdc6 were removed to improve sample behavior for cryo-EM. The color scheme is maintained throughout the figures unless noted otherwise. **b** Purification of *Dm*ORC·DNA·Cdc6 for cryo-EM. Chromatograms are shown for the ternary complex (solid lines) and for isolated DNA (dashed lines). Peak fractions retain all *Dm*ORC subunits and *Dm*Cdc6 when analyzed by SDS-PAGE (inset). The asterisk corresponds to a degradation product of Orc2 and/or Orc3. **c** Side view of the unsharpened cryo-EM map with AAA+ and WH domains colored differentially, highlighting the two-tiered organization of the complex. **d** Near-planar arrangement of the AAA+ and WH modules with respect to the main DNA axis (gray line) in the ORC·Cdc6 ring. The centers of mass of the AAA+ base and WH domains are depicted as spheres. **e** AAA+ and WH view of the cryo-EM density (unsharpened) with ORC and Cdc6 subunits colored differentially. The gap between Orc3 and Orc2 in the WH layer is indicated. **f**, **g** Conformational rearrangements in *Dm*ORC during the transition from the autoinhibited to the activated state. In **f** the positions of the Orc1 AAA+ and Orc2 WH domains in the autoinhibited *apo*-*Dm*ORC state (shown as cartoon; PDB 4xgc^16^) and the ORC·DNA·Cdc6 complex (ODC; depicted as molecular surface) are compared after structural alignment of both assemblies. Arrows indicate the domain movements during activation. Side views are shown, and other subunits/domains are displayed as light gray cartoon. **g** ATP binding to Orc1 reorients the Orc1 AAA+-lid and closes the Orc1/4 interface. The Orc1 AAA+-base subdomain of Orc1 in *apo-Dm*ORC (PDB 4xgc^16^) was superposed on the same region in the ternary complex. BAH, bromo-adjacent homology domain; IDR, intrinsically disordered region; BP, basic patch; WH, winged-helix; NTD, N-terminal domain; CTD, C-terminal domain; TFIIB, transcription factor IIB-like domain. Source data are provided as a Source data file.

For structural studies, we reconstituted the *Dm*ORC·DNA·Cdc6 complex on a 60 bp AT-rich DNA duplex in the presence of ATP using N-terminally trimmed Orc1 and Cdc6 and purified the assembly by size exclusion chromatography (Fig. 2a, b). Cryo-EM analysis of the purified ternary complex revealed uniform particles in different orientations, with secondary structure elements clearly visible in 2D class averages (Supplementary Fig. 2a, b). The majority of complexes retained all components and allowed us to determine the 3D structure of the ternary *Dm*ORC·DNA·Cdc6 assembly at an overall resolution of 3.4 Å (Supplementary Figs. 2c, d, 3a, b, Supplementary Table 1). The 3D EM map showed sufficient detail to generate atomic models for protein components, with Cdc6 being built completely de novo (Supplementary Figs. 3c–f, Supplementary Table 1). The Orc2 N-terminus and the Orc6 TFIIB domain, both of which were disordered or flexible in the cryo-EM map, constitute notable exceptions. The DNA backbone is traceable for ~30 bp of the duplex; however, we were not able to unambiguously assign a DNA sequence to the density, most likely because DNA is bound to ORC in different registers (Supplementary Fig. 3g).

### A near-planar *Dm*ORC·Cdc6 ring encircles and bends DNA

In the DNA-bound initiator·co-loader complex, *Dm*ORC and *Dm*Cdc6 form a two-tiered hexameric ring, with each layer comprising the Orc1–5/Cdc6 AAA+ ATPase and WH domains, respectively (Fig. 2c, d, Supplementary Movie 1). The DNA passes through the center of the complex and is bent at the winged-helix surface toward the Orc3 insert (Fig. 2c–e). While the ATPase ring is closed, the WH tier contains a visible gap between Orc2 and Orc3 (Fig. 2e). In the *Drosophila* complex, both tiers are planar and oriented perpendicular to the DNA axis within ORC’s central channel (Fig. 2d). Comparison of the ternary complex structure with the previously determined crystal structure of *apo-Dm*ORC^16^ reveals that Orc1 has transitioned from the autoinhibited conformation, in which the Orc1 AAA+ module is disengaged from its partner subunit Orc4 and laterally blocks ORC’s central channel, into an active conformation (Fig. 2f). The establishment of canonical AAA+/AAA+ interactions between Orc1 and Orc4, which are similar to those seen in the crystal structure of a subcomplex of human ORC^17^, is facilitated by two distinct conformational changes in Orc1: (a) rotation of the entire Orc1 AAA+ domain and (b) an ATP-induced reorientation of the helical lid subdomain with respect to the AAA+ -base (Fig. 2f, g). The latter rearrangement closes the Orc1/4 AAA+/AAA+ interface by enabling interactions between the Orc1 AAA+-lid and the Orc4 AAA+-base (increasing the buried solvent accessible area from 846 Å^2^ to 1412 Å^2^), rationalizing why ATP binding to ORC stabilizes the active state (Fig. 2g). Besides Orc1, the WH domain of Orc2 has also repositioned in the ORC·DNA·Cdc6 complex and packs against the AAA+ module of Cdc6, propagating the domain-swap observed between AAA+ and WH domains of adjacent protomers around the ORC ring (Fig. 2e, f).

The co-loader Cdc6 is phylogenetically most closely related to ORC’s Orc1 subunit^15^ and adopts a similar 3D structure as Orc1 in the ternary initiator·co-loader complex (Supplementary Fig. 4a). In *Dm*ORC·DNA·Cdc6, the co-loader occupies the position between Orc1 and Orc2 and engages in canonical and non-canonical AAA+/AAA+ interactions with Orc1 and Orc2, respectively, to close the ORC ring (Supplementary Fig. 4c). The N-terminus of Orc3, part of which folds into a β-strand that abuts the central β-sheet of the Orc2 AAA+ core, is sandwiched in between the Orc2 and Cdc6 AAA+ domains, sealing the ATPase ring and forming a tripartite interface (Supplementary Fig. 4b, c). Two conserved Cdc6 arginines positioned in a helix that typically harbors the arginine finger in AAA+ ATPases—a residue involved in stimulating ATP hydrolysis of the adjacent protomer—contribute to the interaction network, indicating that this region in Cdc6 has been co-opted to mediate protein-protein interactions at the non-catalytic Orc2/Cdc6 site (Supplementary Fig. 4c, d).

### DNA recognition by *Dm*ORC·Cdc6

**Fig. 3:**
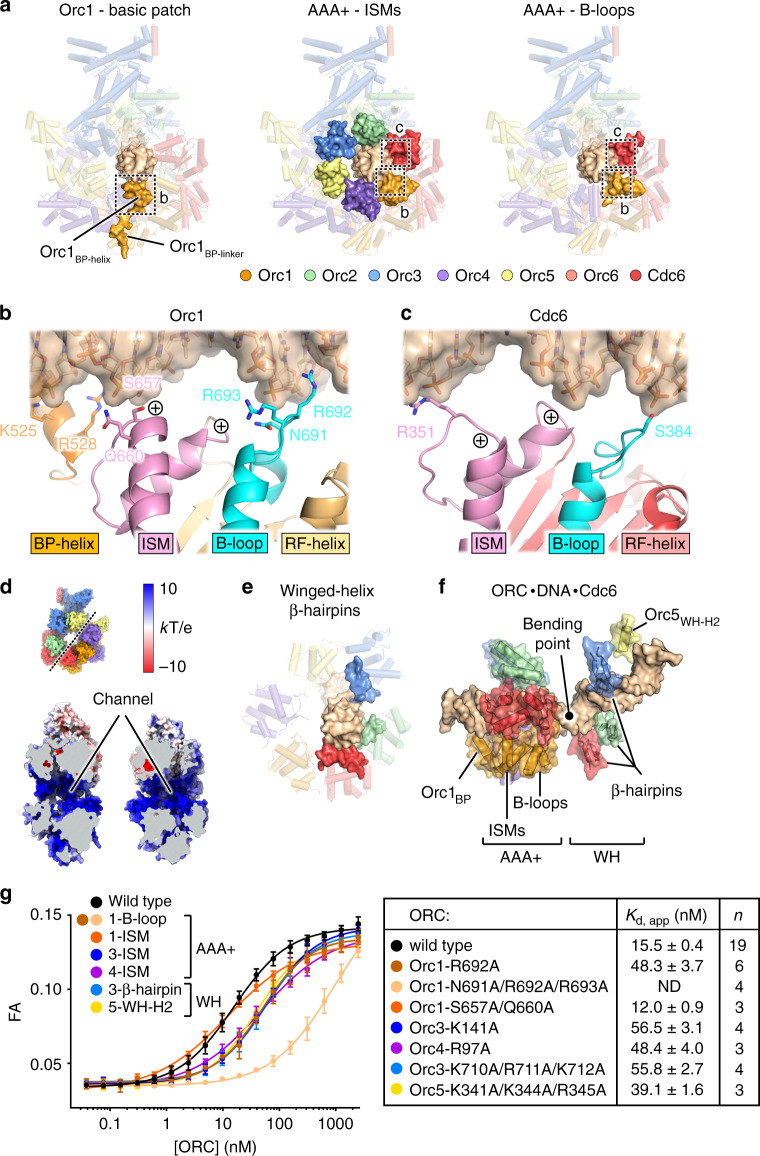
Protein–DNA contacts in the ternary *Dm*ORC·DNA·Cdc6 assembly. **a** The Orc1-basic batch, the ISM ring, and the B-loops of Orc1 and Cdc6 (all shown as molecular surfaces) form three layers within the AAA+ tier that interact with DNA. Close-up views of the Orc1·DNA and Cdc6·DNA interfaces corresponding to boxed areas in (**a**) are displayed in (**b**, **c**), respectively. Amino acid side chains in contact with DNA are shown as sticks, and the positive dipoles of the ISM helices are marked. **d** Open-book view of the *Dm*ORC·Cdc6 channel colored by electrostatic surface potential. The opening plane is noted as a dashed line in the upper overview image of the complex. **e** The β-hairpins of Orc2, Orc3, and Cdc6 (all shown as molecular surface) bind DNA in the WH-tier. The other WH regions are depicted as transparent cartoon. AAA+ domains are omitted for clarity. **f** Summary of protein–DNA contacts in *Dm*ORC·DNA·Cdc6. Only regions that engage DNA are shown. In addition to a subset of the WH β-hairpins, helix 2 of the Orc5 WH domain (Orc5_WH-H2_) is also positioned close to the duplex. Note that in the binary *Dm*ORC·DNA complex, the WH domain of Orc2 does not contact DNA (see Supplementary Fig. 6e). **g** Mutations in DNA binding regions decrease *Dm*ORC’s affinity for DNA as measured by fluorescence anisotropy in the presence of ATP. The means and standard deviations, as well as fitted binding curves, for wild type and mutant ORC assemblies are shown. Amino acid substitutions, the apparent dissociation constants (*K*_d, app_ ±S.E.) for DNA binding, and numbers (*n*) of independent replicates are summarized. ND – *K*_d, app_ could not be determined since binding curves did not reach saturation. Source data are provided as a Source data file.

A distinctive feature of the ternary *Dm*ORC·DNA·Cdc6 assembly is a large channel in the center of the complex that is occupied by DNA (Fig. 2e). Inspection of the cryo-EM density reveals that the DNA is positioned off-center within the *Dm*ORC·Cdc6 channel, arguing that the various subunits differentially contribute to DNA binding in the *Drosophila* complex (Fig. 2e, Supplementary Movie 1). In the AAA+ layer, the DNA closely abuts the ATPase domains of Orc1, Orc4, and Cdc6. This placement is stabilized by three different types of elements that directly engage the duplex (Fig. 3a). The initiator-specific motifs (ISMs) of *Dm*Orc1–5 and Cdc6, a helical insertion in the AAA+ fold characteristic for members of the initiator clade of AAA+ ATPases^10,33^, form a circular collar around DNA. The ISMs of Orc1, Orc4, and Cdc6 form several hydrogen bonds and van der Waals contacts with the sugar-phosphate backbone of one of the DNA strands (Fig. 3b, c, Supplementary Fig. 5). Conversely, the ISMs of Orc2 and Orc3 make more limited contacts with DNA, while the ISM of Orc5 does not directly engage the DNA duplex (Supplementary Fig. 5). Nonetheless, these ISMs are still expected to contribute to DNA binding through the positive N-terminal dipoles of their parallel ISM helices and basic amino acid side chains, which point toward the central channel and add to its electropositive surface potential (Fig. 3d). Unexpectedly, the ISM collar is flanked on either side by additional DNA binding regions. On the outer surface of the AAA+ tier, a basic patch element in Orc1 (preceding the Orc1 AAA+ domain, Fig. 2a) folds into a short α-helix and binds the DNA backbone (Fig. 3a, b, Supplementary Fig. 5). Behind the ISM collar (when viewed from the Orc1 basic patch), Orc1 and Cdc6 each use an additional loop region (referred to as B-loop hereafter) to engage DNA (Fig. 3a–c, Supplementary Fig. 5). In Orc1, the side chain of B-loop arginine 692 inserts into the minor groove where it is stabilized by interactions with the deoxyribose and by the negative electrostatic potential of the backbone phosphate groups but makes no contacts with the nucleobases (Fig. 3b, Supplementary Figs. 3d, 5). Together with the ISMs and the Orc1 basic patch, these B-loops form an extended DNA binding site that promotes the off-center position of the DNA duplex in ORC·Cdc6′s channel.

In the ORC·Cdc6 WH tier, several subunits are also seen to bind DNA (Fig. 3e, Supplementary Fig. 5). Three of them—Orc2, Orc3, and Cdc6—engage the duplex using a β-hairpin element, a signature motif of winged-helix domains that often inserts into the minor groove as exemplified by the related archaeal initiator Orc1/Cdc6^6,7^. However, in the ternary *Dm*ORC·DNA·Cdc6 complex, only the β-hairpin of Orc3 is poised to participate in minor groove interactions (Supplementary Fig. 3e). By contrast, the β-hairpins in Orc1 and Orc4 do not directly engage DNA, while Orc5 contacts the DNA backbone in a non-canonical manner using basic side chains in helix H2 of the WH fold (Fig. 3f, Supplementary Fig. 5).

Binding of the ATPase and WH domains in *Dm*ORC·Cdc6 to DNA leads to significant bending of the DNA duplex by approximately 46 degrees, with a bending point between the AAA+ and WH rings (Figs. 2d, 3f). The curved DNA segment is flanked on either side by the minor-groove-binding Orc1 B-loop and Orc3 β-hairpin, interactions that likely stabilize DNA bending. Contacts between the Orc5 WH-H2 helix and DNA may additionally contribute to duplex remodeling. Importantly, we observe a similar DNA configuration and DNA interactions in a ~3.9 Å resolution cryo-EM structure of a binary *Dm*ORC·DNA complex reconstituted in the absence of Cdc6, indicating that Cdc6 binding does not substantially remodel ORC–DNA contacts (Supplementary Fig. 6). The main difference between both structures pertains to the Orc2 WH domain, which is not visible in *Dm*ORC·DNA (Supplementary Fig. 6e). By contrast, more extensive remodeling is observed in the ORC ring during DNA binding to the initiator when comparing structures of DNA-free, active *Dm*ORC and *Dm*ORC·DNA (Supplementary Fig. 6). Taken together, these structures reveal that multivalent protein/DNA contacts position the duplex in the central substrate-binding ORC channel, whereby the AAA+ domains exert a firm grip on the DNA, and both AAA+ and WH domains cooperate to induce DNA bending (Fig. 3f).

*Dm*ORC binds DNA with low nanomolar affinity in an ATP-dependent manner^18,34^. To understand how the different DNA-binding sites in *Dm*ORC·DNA and *Dm*ORC·DNA·Cdc6 contribute to ORC’s affinity for DNA, we substituted single or multiple conserved residues in these regions with alanine and measured *Dm*ORC’s ability to associate with DNA using a fluorescence anisotropy-based binding assay. As Cdc6 binding to *Dm*ORC·DNA does not alter how the initiator engages DNA (Supplementary Fig. 6), these binding assays report on ORC–DNA contacts in both the binary and ternary complexes. All mutant *Dm*ORC assemblies purified as a stable hexamer, indicating that the substitutions did not interfere with complex assembly (Supplementary Fig. 7a). As seen previously^18^, wild type *Dm*ORC bound a fluorescein-labeled 40 bp AT-rich DNA duplex with an apparent dissociation constant (*K*_d, app_) of ~16 nM in the presence of ATP (Fig. 3g). Alanine substitutions of conserved basic amino acids in the ISMs of Orc3 (K141) or Orc4 (R97) slightly reduced ORC’s affinity for DNA by approximately 3-fold (Fig. 3g). Surprisingly, a double mutation of Orc1-ISM residues S657 and Q660, which engage in van der Waals and hydrogen bond interactions with the DNA backbone, did not impede *Dm*ORCs ability to bind DNA. Unlike Orc3-K141 and Orc4-R97, these Orc1-ISM side chains cannot form salt bridges with the DNA phosphates, rationalizing the disparate findings (Fig. 3g, Supplementary Fig. 5). However, alanine substitutions in the Orc1 B-loop motif had a more striking effect. A triple alanine mutant (Orc1^N691A/R692A/R693A^) completely abolished ATP-dependent, high-affinity DNA binding by *Dm*ORC, which could not be restored by the addition of Cdc6 (Fig. 3g, Supplementary Fig. 5, Supplementary Fig. 7b). By comparison, the β-hairpin of Orc3 and the WH-helix 2 of Orc5 only moderately contribute to stabilizing *Dm*ORC on DNA, with triple mutations of basic residues (Orc3^K710A/R711A/K712A^ or Orc5^K341A/K344A/R345A^) decreasing *Dm*ORC’s affinity for DNA by only 2–3 fold (Fig. 3g). These biochemical data are congruent with the multivalent protein/DNA interactions observed in both *Dm*ORC·DNA and *Dm*ORC·DNA·Cdc6 cryo-EM structures, and also uncover the B-loop in Orc1 as a DNA binding element that is essential for high-affinity interactions of *Dm*ORC with DNA in the absence and presence of Cdc6.

### DNA-induced rearrangements in Orc1 regulate ATP hydrolysis

**Fig. 4:**
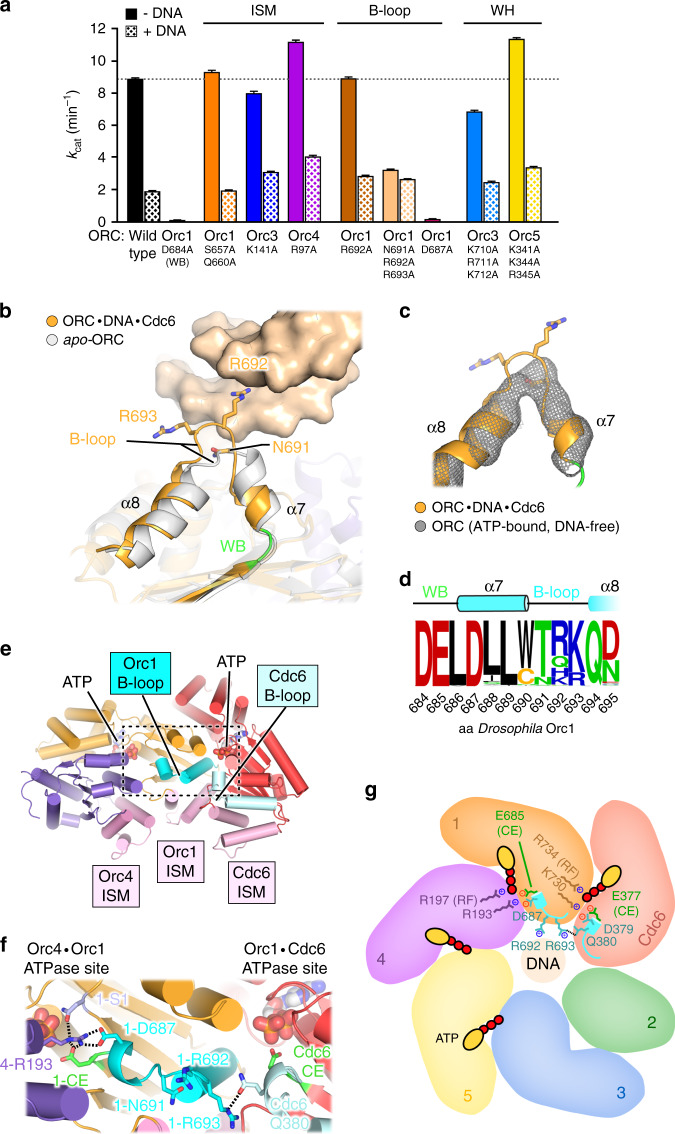
The Orc1 B-loop helps couple DNA binding and ATPase activities in *Dm*ORC. **a** Basal (−DNA) and DNA-inhibited (+DNA) ATPase activities of wild type *Dm*ORC and complexes harboring alanine substitutions of DNA-interacting residues. Mutations in the B-loop of Orc1 substantially reduce *Dm*ORC’s basal ATPase rate and abrogate further inhibition by DNA. Substitutions of Orc1-D684 in the Walker B motif and of Orc1-D687, which links the Orc1 B-loop to active site residues, render the complex catalytically inactive. ATP hydrolysis rates from three independent ATP titrations (except for wild type ORC (*n* = 10 −DNA; *n* = 4 +DNA) and Orc1^R692A^ (*n* = 4 −DNA, *n* = 4 +DNA)) were fit to the Michaelis–Menten equation, and *k*_cat_ and S.E. of fits are plotted as bar graph. **b**, **c** Conformational rearrangement of the Orc1 B-loop upon DNA binding. **b** The Orc1 B-loops of DNA-free *apo*-*Dm*ORC (gray; PDB 4xgc^16^) and the ternary *Dm*ORC·DNA·Cdc6 complex (orange) adopt different configurations. Orc1-R692 inserts into the DNA minor groove. A short α-helix (α7) connects the DNA-binding B-loop to the Walker B motif (green). **c** A comparison of Orc1 B-loop conformations in the ternary complex (orange cartoon) and in ATP-bound, DNA-free ORC (unsharpened EM map depicted as gray mesh). **d** Sequence frequency logo of metazoan Orc1 shows the conservation of B-loop residues and identifies an invariant aspartate (Orc1-D687) between the Walker B motif and the helix preceding the B-loop. Secondary structure features are illustrated above the sequence logo. **e** The B-loops and adjacent helices of both Orc1 and Cdc6, but not their ISMs, are located near the Orc1/4 and Cdc6/Orc1 ATPase sites. AAA+ domains of Orc1, Orc4, and Cdc6 are shown, viewed from ORC’s central channel. **f** Zoomed view of dashed region in (**e**) illustrates the bonding network between the Orc1 B-loop region, adjacent helices, and Orc1/4 active site residues. Dashed lines indicate hydrogen bonds and salt bridges. **g** Summary of interactions from (**f**). The Orc1-sensor 1 residue, which also bonds with Orc4-R193, is omitted for clarity. WB, Walker B (green); CE, catalytic glutamate (green); S1, sensor 1 (light blue); RF, arginine finger (colored by subunit). Source data are provided as a Source data file.

Several ORC subunits and Cdc6 are known to form bipartite ATP binding sites at AAA+/AAA+ interfaces in the ORC and ORC·Cdc6 rings^16,17,20,21,35–37^. In agreement with these studies, we observe clear densities for ATP bound to Orc1/4, Orc4/5, Orc5/3, and Cdc6/Orc1 in our ternary *Dm*ORC·DNA·Cdc6 complex structure (Supplementary Fig. 8). Of these sites, only those formed by Orc1 and Orc4, as well as by Cdc6 and Orc1, retain catalytic activity in yeast and metazoans^17,34,35,37–39^. Notably, ATP hydrolysis by ORC (i.e., at the Orc1/4 interface) is inhibited by DNA^34,35^, but how duplex binding is sensed by ORC and transmitted to the Orc1/4 ATPase site is unknown. To better understand how both events are coupled, we measured the ATP hydrolysis rates of wild type *Dm*ORC and our various DNA binding site mutants in the absence and presence of DNA (Fig. 4a). For wild type *Dm*ORC, we observe a 5-fold decrease in ATP turnover upon DNA addition, a reduction comparable to that reported for *S. cerevisiae* ORC^35^. Amino acid substitutions in the ISMs and WH domains of *Dm*ORC did not drastically alter the basal ATPase rate and still supported DNA-mediated inhibition. Strikingly, the ATP turnover rate of *Dm*ORC carrying a triple alanine mutation in the Orc1 B-loop motif was reduced by ~3-fold and was not attenuated further by DNA, indicating the B-loop may be involved in regulating ATP hydrolysis at the Orc1/4 ATPase site.

Insights into a possible relay mechanism become apparent by comparing the Orc1 B-loop conformation in the ternary *Dm*ORC·DNA·Cdc6 complex with that in the DNA-free *apo-Dm*ORC crystal structure and our DNA-free ATP-*Dm*ORC cryo-EM structure^16^. In the presence of DNA, the Orc1 B-loop adopts a more extended configuration, which leads to partial unwrapping of a short α-helix that connects the B-loop to the catalytic Walker B motif (Fig. 4b, c). Interestingly, the B-loop residues and the preceding α-helix are highly conserved in metazoan Orc1 proteins (Fig. 4d). An invariant aspartate (D687) in this helix is part of a bonding network that positions the sensor 1 (a residue required for ATP hydrolysis in AAA+ ATPases) and likely also the catalytic glutamate (a conserved amino acid that helps activate the lytic water for catalysis) in the Orc1/4 ATPase site (Fig. 4e–g). Importantly, an Orc1^D687A^ mutation abolishes ATP hydrolysis by *Dm*ORC, supporting a critical role for the pre-B-loop helix in organizing this ATPase center (Fig. 4a). We propose that the Orc1^N691A/R692A/R693A^ mutant alters the B-loop conformation in a manner that impedes both DNA binding and ATP-hydrolysis (Figs. 3g, 4a). Although higher resolution structures will be required to resolve the exact rotamer conformations of residues involved in the allosteric coupling, these experiments nonetheless uncover a critical role for the Orc1 B-loop not only in DNA binding, but also in regulating ATP hydrolysis by ORC. A comparable relay mechanism might also extend to Cdc6, where the B-loop likewise contacts DNA and connects to the Cdc6/Orc1 ATPase center via a similar bonding network as seen for Orc1 (Fig. 4e, f). Interactions between the Orc1 and Cdc6 B-loops and neighboring residues could additionally help regulate the temporal order of ATP hydrolysis at both ATPase sites (Fig. 4f, g).

### Comparison of DNA binding in *DmORC* and *Sc*ORC

**Fig. 5:**
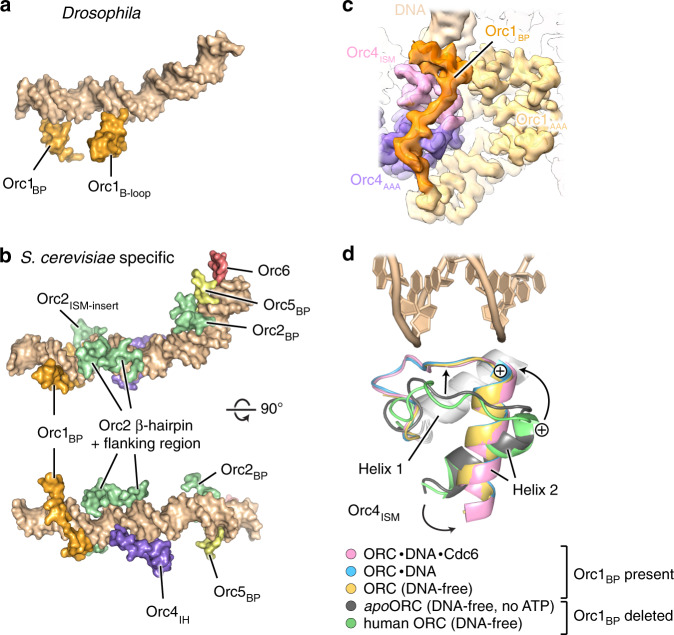
Comparison of *S. cerevisiae* and *Drosophila* ORC–DNA contacts. **a** The B-loop of Orc1 engages DNA in *Drosophila* but not in budding yeast ORC, while interactions between the Orc1-BP and DNA differ in both systems. DNA binding elements shared with *S. cerevisiae* ORC are not shown. **b** *S. cerevisiae* ORC uses numerous additional regions to associate with DNA in the cryo-EM structure of the DNA-bound budding yeast initiator (PDB 5zr1^20^). A subset of these are yeast-specific insertions in ORC subunits (e.g., Orc2_ISM-insert_, Orc4_IH_), while others are flexible in *Dm*ORC·DNA and/or *Dm*ORC·DNA·Cdc6 (i.e., Orc2_BP_, Orc5_BP_, Orc6_TFIIB_). Only duplex-binding regions unique to the *S. cerevisiae* structure are shown. Note also that the basic patch of *S. cerevisiae* Orc1 tracks in the DNA minor groove. **c** In *Drosophila* Orc1, the basic patch packs against the ISM of Orc4 and N-terminally folds into a helix that contacts the phosphodiester backbone but does not engage the minor groove. Unsharpened EM map density is shown. **d** Docking of the Orc1-basic patch reorients the second Orc4-ISM helix, priming it for DNA binding. The Orc4-ISMs from *Dm*ORC structures determined in this study (all containing the Orc1-basic patch) are superposed onto the Orc4-ISMs from the *apo*-*Dm*ORC and human Orc1/4/5 crystal structures (PDBs 4gxc^16^ and 5uj7^17^; both determined in the absence of the Orc1-basic patch). The repositioning of the positive ISM helix 2 dipole is indicated. BP, basic patch; IH, insertion helix; ISM, initiator-specific motif.

Among eukaryotic initiators, the sequence-specific recognition of DNA consensus motifs is a unique property of ORC in *S. cerevisiae* and a few related *Saccharomycetes spp*., while metazoan ORC assemblies bind DNA in a sequence-independent manner^14,25,30,40,41^. Our structure of the ternary *Dm*ORC·DNA·Cdc6 complex afforded comparative analysis with a recent cryo-EM model of DNA-complexed *S. cerevisiae* ORC (*Sc*ORC)^20^ to unveil the physical basis for these divergent DNA binding modes. Common features of both initiators include DNA bending and the use of the ISM collar and the Orc3 WH β-hairpin for DNA binding, albeit how these elements interact with nucleic acid varies slightly. Nonetheless, numerous differences in ORC/DNA contacts are also apparent (Fig. 5a, b, Supplementary Movie 1). In the *Sc*ORC·DNA complex, the Orc1 B-loop is pushed away from DNA by the Orc2 WH β-hairpin, suggesting it is a DNA binding element unique to metazoans. Conversely, many additional regions of *Sc*ORC, but not *Dm*ORC, interact with DNA, which results in a substantially larger solvent-accessible surface area that is buried upon DNA binding of the budding yeast initiator (~3900 Å^2^ for *Sc*ORC vs. ~1200 Å^2^ for *Dm*ORC) (Fig. 5b). Notably, the TFIIB domain of Orc6, the C-terminal part of which binds DNA in *Sc*ORC^20^, is not visible in the binary or ternary DNA-bound *Dm*ORC assembly, even when the length of the DNA duplex is increased (Fig. 5b, Supplementary Fig. 9a–c). This disparity can be explained by the lack of a yeast-specific loop insertion in the WH domain of Orc5, which in *Sc*ORC likely helps position the C-terminal TFIIB module near the DNA duplex (Supplementary Fig. 9d–f). Basic patches in Orc2 and Orc5 that cooperate with Orc6 to achieve DNA bending in *Sc*ORC·DNA are likewise not ordered in DNA-bound *Dm*ORC assemblies (Fig. 5a, b, Supplementary Fig. 9d, e); these observations suggest that the corresponding *Dm*ORC regions are not strictly required for inducing DNA bending, although they could become important during later steps of Mcm2–7 loading and help stabilize the duplex segment distal of the bend.

Another important difference relates to the role of the Orc1 basic patch (Orc1-BP) in DNA binding. Mutation of this element in *Dm*ORC and *Sc*ORC attenuates the affinity of both initiators for DNA^18,42^, but the underlying structural mechanism for these concordant observations is strikingly different. Instead of snaking along the minor groove as seen in *Sc*ORC·DNA^20^, the N-terminal part of *Dm*Orc1′s basic patch folds into a helix that only locally interacts with the sugar-phosphate backbone (Figs. 3b, 5a–c, Supplementary Fig. 5). In addition, this *Dm*Orc1-BP helix and the adjacent linker also pack against the ISM of Orc4 (Fig. 5c). Comparing *Drosophila* and human ORC structures obtained in the absence or presence of the Orc1 basic patch reveals that these interactions trigger a rotation of the second Orc4-ISM helix, moving the positive, N-terminal Orc4 helix dipole closer to DNA (Fig. 5d). Therefore, the strong DNA binding defect of *Dm*ORC seen previously upon deleting the Orc1 basic patch is likely caused by the combined effect of abrogating Orc1-BP contacts with DNA and the allosteric influence on the Orc4-ISM^18^. At the same time, the paucity of interactions with the DNA grooves, which in *Sc*ORC are involved in base-pair recognition of ARS consensus sequences^20^, rationalizes the reported sequence promiscuity of *Dm*ORC and orthologous initiators in metazoans, and most likely also within the broader eukaryotic domain. Taken together, our results show that budding yeast and metazoan initiators share a subset of common DNA binding regions but have also evolved specialized elements that suit the specific needs of these initiators in origin recognition.

### DNA geometry and malleability modulate *Dm*ORC activities

**Fig. 6:**
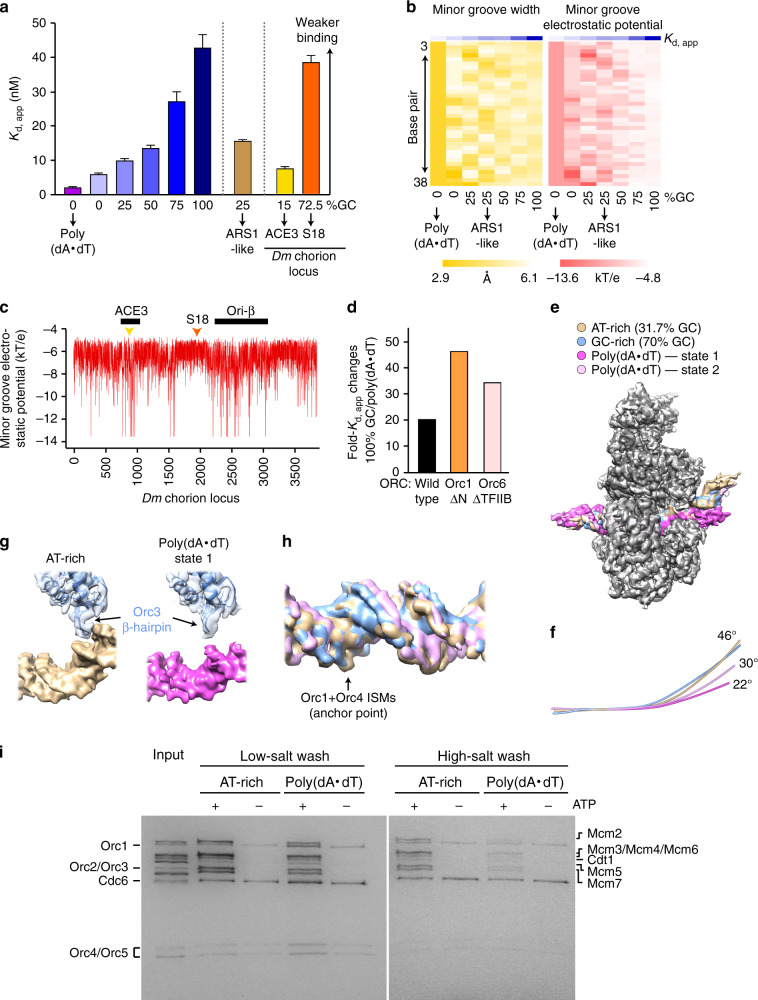
DNA sequence influences DNA binding and remodeling activities of *Dm*ORC. **a** Increasing GC-content alleviates *Dm*ORC’s ATP-dependent affinity for DNA. The apparent dissociation constants (*K*_d, app_) and standard errors (S.E.) of fit for ATP-dependent *Dm*ORC binding to different DNA duplexes is summarized in a bar graph (see Supplementary Fig. 10a for corresponding binding curves and number of replicates). The ARS1-like duplex used in Fig. 3g is shown for comparison. ACE3 and S18 are endogenous DNA sequences of the *Drosophila* chorion locus. **b** Predicted minor groove width and electrostatic potential of DNA duplexes used in (**a**) are plotted for each base pair to highlight the correlation with measured *K*_d, app_ for *Dm*ORC binding. **c** Minor groove electrostatic potential across the *Drosophila* chorion locus. The ORC-binding ACE3 and ori-β regions are highlighted by black bars. The positions of the endogenous DNA sequences used in (**a**) are indicated by triangles. **d** Deletion of secondary DNA binding sites in *Dm*ORC enhances *Dm*ORC’s preference for associating with AT-rich duplexes. The ratios of *K*_d, app_-100% GC over *K*_d, app_-poly(dA·dT) are plotted for wild type, full-length *Dm*ORC, and complexes lacking respective secondary DNA binding regions. See Supplementary Fig. 10a−c for binding curves. **e** Superposition of cryo-EM maps (unsharpened) of *Dm*ORC and *Dm*Cdc6 (all colored in gray tones) complexed with different DNA duplexes. The structure of the AT-rich (31.7% GC) assembly corresponds to the one shown in previous figures. **f** DNA axes of structures in **e** illustrate different degrees of DNA bending by *Dm*ORC. **g** Diminished DNA bending precludes minor groove engagement by the Orc3 WH β-hairpin. **h** Variability of DNA backbone positions within the AAA+ ring. EM densities (locally sharpened with LocScale) for DNA duplexes in the different *Dm*ORC·DNA·Cdc6 structures are shown (except for state 1 of the poly(dA·dT) complex). **i** DNA sequence modulates Mcm2–7 loading efficiency. *Dm*Mcm2–7 is recruited (low-salt wash) similarly to AT-rich and poly(dA·dT) duplexes but loaded (high-salt wash) more efficiently onto AT-rich DNA compared to poly(dA·dT) duplexes. Note that Cdt1 and Cdc6 nonspecifically associate with beads. Source data are provided as a Source data file.

Although *Dm*ORC engages DNA predominantly through DNA-sequence-independent backbone contacts in our structures, human and *Drosophila* ORC have been reported to prefer to bind AT-rich DNA^25,30^, but the physical basis for these observations is unknown. Given the paucity of DNA base-contacts by *Dm*ORC, we hypothesized that the affinity differences for dissimilar DNA duplexes may result from sequence-dependent differences in DNA duplex geometry. Therefore, we systematically determined the dissociation constants for *Dm*ORC and DNA duplexes of varying GC-content and correlated the measured affinities with predicted DNA shape parameters. We found that *DmORC* binds more tightly to AT-rich sequences, with an overall 20-fold difference in apparent *K*_d_ between homopolymeric poly(dA·dT) duplexes and duplexes of 100% GC-content, a range larger than observed previously for *Dm*ORC^25^ (Fig. 6a, Supplementary Fig. 10a). Importantly, the apparent *K*_d_ values of *Dm*ORC binding to DNA did not only correlate with GC-content, but also with minor groove width and, more pronouncedly, with minor groove electrostatic potential, suggesting that thermodynamic duplex properties modulate ORC–DNA interactions (Fig. 6b). A similar relationship is observed when using endogenous *Drosophila* DNA sequences; known ORC binding sites at the well-studied *Drosophila* chorion locus, such as ACE3, are characterized by increased negativity of the minor groove electrostatic potential (Fig. 6a, c)^43^. The robust nucleotide-dependence of DNA binding by *Dm*ORC in our assay conditions (>100-fold tighter DNA association in the presence of ATP^18^) argues that the varying affinities for different DNA sequences are mediated by binding elements in the ORC core rather than secondary DNA binding sites. Concordantly, deletion of secondary DNA binding sites, which include the Orc6 TFIIB domain or the long Orc1 N-terminus^23,32^, enhances *Dm*ORC’s ability to discriminate between duplexes of high and low GC content (Fig. 6d, Supplementary Fig. 10a–c).

To understand the structural mechanisms for *Dm*ORC’s preferential binding to AT-rich DNA and its consequences for ORC-dependent DNA remodeling, we reconstituted two additional *Dm*ORC·DNA·Cdc6 complexes, using poly(dA·dT) dsDNA and a 70% GC duplex (referred to as GC-rich hereafter), respectively, and determined their structures by cryo-EM. The overall resolution of these structures was with 3.2 Å and 3.4 Å comparable to that of the *Dm*ORC·DNA·Cdc6 structure containing AT-rich dsDNA (GC content of 31.7%) (Supplementary Fig. 10d–f). Unexpectedly, we identified two different DNA conformations in our poly(dA·dT) dataset, which are characterized by different bending angles (~22° in state 1 vs. ~30° in state 2), and both are bent by *Dm*ORC to a lesser extent than the AT-rich duplex (46° bend) (Fig. 6e, f). The reduced bending of poly(dA·dT) DNA in the ternary complex probably results from an increased rigidity of the duplex that prevents the Orc3 WH β-hairpin to engage the minor groove (Fig. 6g). By contrast, the ORC subunits and Cdc6 adopt very similar configurations when bound to poly(dA·dT) or mixed nucleotide duplexes (Fig. 6e). One exception is the WH domain of Orc2, for which we observe weaker density when the DNA is bent to only 22° (state 1 of the poly(dA·dT)-containing complex), suggesting that a reduced curvature of DNA interferes with docking of this module onto the ORC·Cdc6 AAA+ ring (Supplementary Fig. 10d). The GC-rich DNA, on the other hand, assumes a conformation that globally resembled the AT-rich duplex (Fig. 6e, f). Nonetheless, closer inspection of the different DNA densities revealed variations in the position of the phosphodiester backbone, especially within the AAA+ ring where the DNA densities are best resolved. While the phosphates of the different DNAs superimpose well near ISMs of Orc1 and Orc4, their position is more variable in other regions (Fig. 6h). Thus, the AAA+ ring is the principal ORC region that stabilizes the *Drosophila* initiator on DNA, with the ISMs of Orc1 and Orc4 serving as an anchor point to position the DNA duplex, while adjacent regions modulate ORC’s affinity for distinct DNA duplexes.

Our structure of DNA-bound *Drosophila* initiator assemblies, together with that of *Sc*ORC·DNA determined previously^20^, suggests that DNA bending is a conserved activity of eukaryotic initiators; yet, whether DNA remodeling is required for Mcm2–7 loading is unknown. To address this question, we examined whether a poly(dA·dT) duplex, which is bound by ORC with high affinity but bent to a lesser degree as the mixed AT-rich duplex (Fig. 6a, e, f), is a substrate for Mcm2–7 loading. Strikingly, using our in vitro reconstitution system for *Drosophila* Mcm2–7 loading we find that Mcm2–7 is loaded onto poly(dA·dT) duplexes much less efficiently than onto the AT-rich substrate (Fig. 6i, high-salt wash). By contrast, Mcm2–7 is recruited to both DNA substrates to similar extents in low-salt conditions (Fig. 6i). Collectively, these biochemical and structural findings argue that sequence-dependent thermodynamic properties of DNA, including duplex geometry and deformability, tune ORC’s ability to productively engage nucleic acid and to load Mcm2–7 onto DNA.

## Discussion

**Fig. 7:**
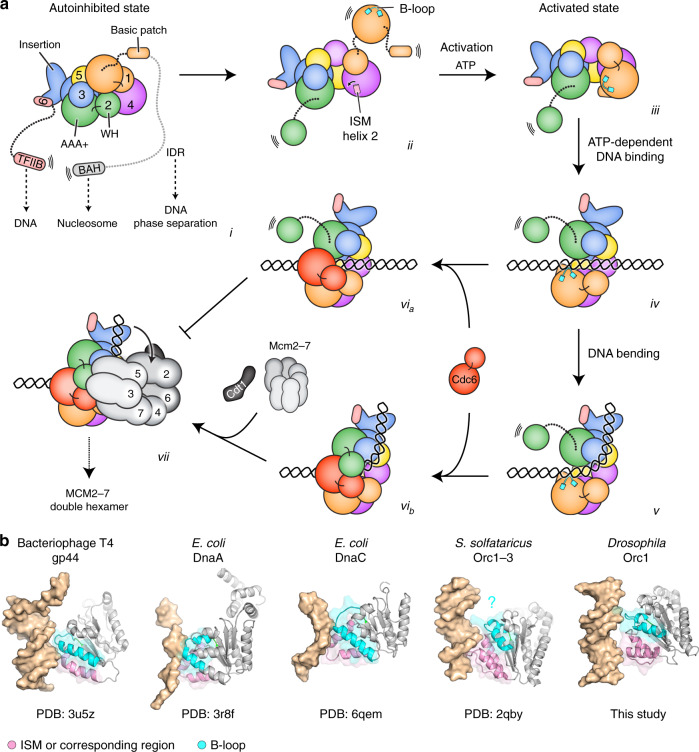
Metazoan ORC activities during Mcm2–7 loading. **a** Summary of conformational transitions in metazoan ORC and of DNA binding and remodeling mechanisms (panels *i-vii*, see text for details). ORC regions that have been implicated in recruiting ORC to origins by serving as secondary DNA binding sites (Orc1-IDR, Orc6-TFIIB) or nucleosome-interacting domains (Orc1-BAH) are indicated. The N-terminal region in Orc1 deleted in our construct used for structure determination is shown in gray. TFIIB, transcription factor IIB-like domain in Orc6; BAH, bromo-adjacent homology domain in Orc1; IDR, intrinsically disordered region. **b** Comparison of DNA contacts in diverse initiator and loader proteins highlights the B-loop element as a common DNA binding region. Archaeal Orc1/Cdc6 constitutes an exception since its B-loop is more distant from DNA compared to the other AAA+ ATPase modules. Nucleic-acid bound AAA+ domain structures of bacteriophage T4 clamp loader (PDB 3u5z^85^), *E. coli* DnaA (PDB 3r8f^12^) and DnaC (PDB 6qem^59^), *S. solfataricus* Orc1-3 (PDB 2qby^6^), and *Drosophila* Orc1 (this study) are shown. The initiator-specific motifs (ISM) or corresponding region in the clamp loader, and B-loop regions are depicted as transparent pink and cyan surfaces, respectively.

The various cryo-EM structures of DNA- and Cdc6-bound *Dm*ORC presented here, in combination with the previous crystal structure of the DNA-free *apo*-complex^16^, afford the direct comparison of different ORC functional states in a single species to define structural transitions in the initiator during early stages of replication initiation. In nucleotide-free *Dm*ORC, the Orc1 AAA+ domain is disengaged from its Orc4 partner, precluding productive DNA binding in ORC’s channel (Fig. 7a, *i–ii*). The first major restructuring event of ORC occurs during ATP binding, which alters the conformation of Orc1′s AAA+ domain to stabilize interactions of this module with the AAA+ domain of Orc4 during ORC activation (Fig. 7a, *iii*). Juxtaposition of the Orc1 and Orc4 ATPase modules facilitates docking of the Orc1 basic patch helix and linker onto Orc4, which in turn triggers a rotation of the Orc4 ISM helix and primes ORC for DNA binding in its central channel. Mutations or deletions of the Orc1-BP reduce *Dm*ORC’s affinity for DNA, supporting the notion that the Orc4-ISM/Orc1-BP platform is critical for DNA engagement^18^. Interestingly, the Orc1-BP harbors two cyclin-dependent kinase (CDK) phosphorylation sites; phosphorylation of *Dm*ORC has been reported to inhibit ATP-dependent DNA binding^44^, an outcome that may be caused by destabilization of the Orc1-BP/Orc4-ISM DNA binding site. Lastly, conformational flexibility of the ORC ring, mediated by motions at the Orc3/Orc5 interface, likely promotes DNA entry into ORC’s channel by transiently widening of the gap between Orc1 and Orc2 (Fig. 7a, *iv–v*). Thus, multiple conformational rearrangements in the initiator, some of which are conserved among eukaryotic ORC assemblies, control its association with DNA. For example, instability of the Orc1/4 AAA+/AAA+ interface in the absence of ATP has also been observed in human and *S. cerevisiae* ORC, offering a structural explanation for the ATP-dependence of ORC/DNA interactions in ORC’s central channel^16,18,20^. Undocking of Orc1 from Orc4 after ATP hydrolysis and/or transitioning into an autoinhibited state could also provide a means to release ORC from DNA or to disengage the complex from Mcm2–7 after loading.

Activation of ORC and DNA binding to the initiator allows the association of the co-loader Cdc6 (Fig. 7a, *vi*). Surprisingly, Cdc6 binding does not remodel ORC–DNA contacts or alter *Dm*ORC’s ATPase activity, nor does it induce major conformational changes in the ORC ring apart from providing an interaction surface for the Orc2 WH domain, which is flexible in Cdc6′s absence. These findings indicate that Cdc6 predominantly has a structural role during Mcm2–7 loading. In agreement with this model, Cdc6 and the Orc2 WH module form a combined binding platform for the Mcm3 WH domain in the OCCM complex, and biochemical studies have found that this Mcm3 region is essential for recruitment of both Mcm2–7 hexamers^21,45–47^. In this regard, it is interesting to note that efficient docking of the Orc2 WH domain against the ORC·Cdc6 ring also requires a certain degree of DNA bending and may, therefore, provide a means to regulate Mcm2–7 recruitment and loading.

Structural comparison of DNA-bound *S. cerevisiae* and *Dm*ORC assemblies reveals that both initiators have evolved a subset of specialized DNA binding elements that reflect the distinct modes of origin recognition in these systems. In budding yeast ORC, DNA interactions facilitate recognition of the ARS consensus sequence at replication origins^20,21^. By contrast, *Dm*ORC and probably all other metazoan initiators are optimized to bind DNA in a sequence-independent manner through DNA backbone interactions with regions of the positively charged ORC central channel. Nonetheless, these contacts, likely in conjunction with the DNA minor groove engagement by a conserved Orc1 B-loop residue (either arginine, lysine or histidine in metazoan Orc1 orthologs), also provide a means for recognition of DNA-sequence-dependent variations in DNA duplex geometry. These association can account for *Drosophila* and human ORC’s preference for binding AT-rich or poly(dA·dT) DNA segments in vitro and are also consistent with an enrichment of these sequence features at chromosomal ORC binding sites and near replication start sites in vivo^25,26,30,43,48,49^. Thus, AT-rich sequences and poly(dA·dT) stretches at metazoan replication origins may not only promote initiation by maintaining nucleosome-free regions^50^, but also by stabilizing metazoan ORC on DNA through high-affinity interactions in ORC’s central channel. These mechanisms are expected to work in concert with chromatin cues (e.g., interactions of the Orc1-BAH domain with H4K20me2 on nucleosomes and other chromatin-associated ORC partner proteins) and secondary DNA binding sites (including the Orc6-TFIIB module and the phase separating IDR in Orc1) to recruit ORC to origins on metazoan chromosomes^22,23,25,27,32,48,49,51–56^. The extent to which these distinct strategies are used in combination at individual origins or may change during development is an important question for future studies.

Both *S. cerevisiae* and *Dm*ORC bend DNA, which has been proposed to help align the DNA duplex with the gate in the Mcm2–7 ring during helicase loading (Fig. 7a, *vii*)^18,20^. Unexpectedly, we observed varying degrees of *Dm*ORC-induced DNA bending of duplexes containing different DNA sequences, demonstrating that DNA binding and bending can be uncoupled. Although ORC’s inability to bend certain DNA sequences, in particularly poly(dA·dT) did not prevent high-affinity DNA binding or co-association of Cdc6, it inhibited Mcm2–7 loading, likely by impeding DNA insertion into the helicase ring (Fig. 7a). Given the importance of DNA bending for efficient Mcm2–7 loading, it is somewhat surprising that DNA bending in our DNA-complexed *Dm*ORC structures is not as extensive as in the *Sc*ORC·DNA assembly, in which the bent duplex is stabilized by yeast-specific ORC/DNA contacts mediated by Orc6 and basic patches in Orc2 and Orc5^20^. However, it is possible that similar interactions are more transient in the *Drosophila* initiator·DNA complexes, or that Mcm2–7 recruitment is necessary to promote more extensive DNA remodeling prior to helicase loading. Collectively, our findings suggest a model in which the location of metazoan Mcm2–7 loading sites, and thereby licensed origins, is in part modulated by DNA-sequence induced variations in DNA geometry and malleability. Binding of metazoan ORC to DNA regions that are more resistant to deformation (such as poly(dA·dT) stretches), may require the initiator to passively slide on DNA until it encounters a malleable DNA segment^30,50,57^. Alternatively, metazoan ORC may recruit specific protein co-factors that help productively bend DNA to increase helicase loading efficiency. In this regard, reconstituting metazoan Mcm2–7 loading in vitro, which we have done here with recombinant *Drosophila* proteins, will provide a useful system to investigate these different possibilities in the future.

Binding of substrates in the central pore of oligomeric AAA+ ATPases often regulates the enzymatic activity of these assemblies^11,58^. In ORC, DNA binding inhibits ATP hydrolysis at the Orc1/4 site, the principal ATPase center, in the yeast and the *Drosophila* complex^34,35^ (Fig. 4a). Our structural and biochemical studies identify the B-loop region in *Drosophila* Orc1 to be involved in sensing and communicating the DNA binding status in ORC to the Orc1/4 ATPase site. Although in the DNA-bound *S. cerevisiae* initiator (unlike in *Dm*ORC·DNA assemblies), the Orc1 B-loop element does not directly contact the DNA duplex, conformational changes in this region may be achieved indirectly upon DNA engagement of yeast-specific DNA binding elements^20^; high-resolution structural information of DNA-free *S. cerevisiae* ORC will be necessary to test this hypothesis. Notably, the corresponding regions in *Drosophila* and budding yeast Cdc6 (in the OCCM complex^21^) directly interact with DNA, suggesting that Orc1 and Cdc6 may use similar strategies to regulate ATP hydrolysis at the Orc1/4 and Cdc6/Orc1 sites, respectively. Inspection of structures of related initiator and loader AAA+ ATPases further revealed that the B-loop region is universally located at the nucleic-acid binding face of these proteins and, in addition to the ISMs, often directly contacts DNA (Fig. 7b). In the bacterial helicase loader DnaC, the B-loop also undergoes a conformational change upon DNA association and is required for ssDNA binding and ATP hydrolysis^59^. This structural congruency suggests that nucleic-acid engagement by the B-loop element may constitute a more general strategy of AAA+ ATPases in the initiator and helicase loader clade to stabilize these proteins on DNA. Future studies will help determine how these interactions are coupled to the ATPase cycle in the different systems to support the diverse biological functions of these molecular switches.

## Methods

### Protein constructs and baculovirus generation

The various *Drosophila* ORC (*Dm*ORC) assemblies used in this study were reconstituted in insect cells by co-infection of baculoviruses expressing either individual or multiple ORC subunits. To generate ORC-containing vectors for baculovirus production, full-length or N-terminally truncated ORC subunits were first cloned into a ligation-independent-cloning (LIC)-compatible pFastBac vector (Macrolab, University of California Berkeley, USA). For purification, a hexa-histidine (6xHis) tag and a maltose binding protein (MBP) tag, both followed by a tobacco etch virus (TEV) protease cleavage site, were added to the N-termini of Orc1 and Orc4, respectively. These constructs served as templates for site-directed mutagenesis to introduce point mutations into Orc1, Orc3, Orc4, and Orc5. All individual wild type and mutant ORC subunit constructs were verified by DNA sequencing. Subsequently, several wild type and mutant ORC subunits were combined into a pFastBac-derived BioBricks MultiBac expression vector (Macrolab, University of California Berkeley, USA) by subcloning different combinations of *Dm*ORC genes. Bacmids of single and MultiBac expression constructs were generated in DH10Bac cells and used for transfections of Sf9 cells with Cellfectin II (Thermo Scientific Fisher). Baculoviruses were amplified for two rounds in Sf9 cells to obtain high-titer viruses for infection of Hi5 cells for large-scale *Dm*ORC expression.

*Drosophila* Cdc6 was also expressed in insect cells. The coding sequences of full-length or N-terminally truncated *Dm*Cdc6 (ΔN241, amino acids 242-662) were cloned into the LIC-compatible pFastBac vector (Macrolab, University of California Berkeley, USA). The full-length *Dm*Cdc6 construct contained an N-terminal MBP tag followed by a TEV cleavage site, while that of *Dm*Cdc6ΔN241 was fused to an N-terminal 6xHis-MBP tag and a TEV cleavage site. Bacmid generation and baculovirus amplification were performed as described for *Dm*ORC.

*Drosophila* Cdt1 and Mcm2–7 were expressed in *E. coli* and Hi5 insect cells, respectively. Full-length or N-terminally trimmed (amino acids 298-743) *Dm*Cdct1 were cloned as N-terminal 6xHis-TEV fusions into a LIC-converted, pET-derived *E. coli* expression vector (Macrolab, University of California Berkeley, USA), while Mcm2–7 subunits were cloned into two pFastBac-derived BioBricks MultiBac expression vectors (Macrolab, University of California Berkeley, USA), one construct containing Mcm2, Mcm4, and Mcm6, and the other Mcm3, Mcm5, and Mcm7. For affinity purification, Mcm4 and Mcm7 were tagged N-terminally with MBP and 6xHis, respectively, each followed by a TEV protease cleavage site.

### Expression and purification of recombinant *Dm*ORC and *Dm*Cdc6

The different *Dm*ORC assemblies were purified at 4 °C from 4 liters Hi5 cells infected with combinations of baculoviruses expressing single or multiple *Dm*ORC subunits as previously^16,19^ with minor modifications. 48 h post-infection, Hi5 cells were harvested, resuspended in ~130 mL lysis buffer (50 mM Tris-HCl, pH 7.8, 300 mM KCl, 50 mM imidazole, pH 7.8, 10% glycerol, 200 μM PMSF, 1 μg/mL leupeptin, 1 mM BME), and lysed by sonication. The crude cell lysate was clarified by two ultracentrifugation runs in a Beckman Coulter Optima L-80 XP ultracentrifuge, each at 142,414 × *g* for 45 min in a 45Ti rotor. After the first centrifugation step, ammonium sulfate was added to the lysate supernatant to a final concentration of 20% (v/v). The supernatant of the second ultracentrifugation run was applied to a 5 mL HisTrap HP nickel-affinity chromatography column (GE Healthcare). After extensive washing with lysis buffer, bound *Dm*ORC was eluted with a 50–250 mM imidazole gradient in 50 mM Tris-HCl, pH 7.8, 300 mM KCl, 10% glycerol, 1 mM BME and further purified using a 10 mL amylose column (New England Biolabs). 6xHis and MBP tags were cleaved by overnight incubation with 6xHis-tagged TEV protease, which was removed by subsequent nickel-affinity chromatography using a 5 mL HisTrap HP column (GE Healthcare). *Dm*ORC was concentrated in a 30K Amicon Ultra-15 concentrator (Millipore) and subjected to gel filtration chromatography on a HiPrep 16/60 Sephacryl S-400 HR column (GE Healthcare) equilibrated in 25 mM HEPES-KOH pH 7.6, 10% glycerol, 1 mM DTT and either 500 mM potassium glutamate (for most biochemical assays), or 300 mM potassium acetate and 10 mM magnesium acetate (for reconstituting DNA·ORC and DNA·ORC·Cdc6 assemblies for cryo-EM). Peak fractions containing *Dm*ORC were pooled and concentrated in 30K Amicon Ultra-15 concentrators (Millipore). Proteins were then aliquoted, flash frozen in liquid nitrogen, and stored at −80 °C.

Full-length MBP-tagged *Dm*Cdc6 and 6xHis-MBP-tagged *Dm*Cdc6ΔN241 were expressed in Hi5 cells for 48 h. For purification of full-length *Dm*Cdc6, cells were resuspended in 100 mL buffer containing 50 mM Tris-HCl, pH 7.8, 600 mM KCl, 10% glycerol, 1 mM DTT, 200 μM PMSF, 1 μg/mL leupeptin. Cell lysis and extract clarification were performed as described for *Dm*ORC. Full-length MBP-tagged *Dm*Cdc6 was purified by amylose affinity chromatography on a 10 mL column (New England Biolabs). After extensive washing with lysis buffer, the salt concentration was lowered to 300 mM KCl and the protein eluted with 20 mM maltose in 50 mM Tris-HCl, pH 7.8, 300 mM KCl, 10% glycerol, 1 mM DTT. *Dm*Cdc6-containing fractions were pooled, concentrated in 30K Amicon Ultra-15 concentrators (Millipore), and the protein was further purified by gel filtration chromatography on a HiPrep 16/60 Sephacryl S-300 HR column (GE Healthcare) equilibrated in 25 mM HEPES-KOH, pH 7.6, 500 mM potassium glutamate, 10% glycerol, 1 mM DTT.

To purify N-terminally trimmed *Dm*Cdc6, Hi5 cells expressing 6xHis-MBP-tagged *Dm*Cdc6ΔN241 were resuspended in 150 mL lysis buffer containing 50 mM Tris-HCl, pH 7.8, 600 mM KCl, 50 mM imidazole, pH 7.8, 10% glycerol, 5 mM magnesium acetate, 1 mM BME, 200 μM PMSF, and 1 μg/mL leupeptin. After clarifying the lysate by ultracentrifugation and 20% (v/v) ammonium sulfate precipitation, *Dm*Cdc6ΔN241 was purified by affinity chromatography using a 5 mL HisTrap HP column (GE Healthcare) and a 10 mL amylose column (New England Biolabs). The protein was eluted from these columns with a 50–250 mM imidazole gradient and 20 mM maltose, respectively, both in 50 mM Tris-HCl, pH 7.8, 600 mM KCl, 10% glycerol, 5 mM magnesium acetate, 1 mM BME. The affinity tags were removed by overnight cleavage with 6xHis-tagged TEV protease and a subsequent nickel-affinity step to remove the 6xHis-MBP tag, uncleaved protein, and TEV protease. The flow-through was concentrated in a 10K Amicon Ultra-15 concentrator (Millipore) and loaded onto a HiPrep 16/60 Sephacryl S-300 HR column (GE Healthcare) equilibrated in 25 mM HEPES-KOH, pH 7.6, 300 mM potassium acetate, 10 mM magnesium acetate, 10% glycerol, 1 mM DTT. Peak *Dm*Cdc6ΔN241 fractions eluting from the gel filtration column were pooled, concentrated in a 10K Amicon Ultra-15 concentrators (Millipore), and flash frozen in liquid nitrogen for subsequent storage at −80 °C. A 6xHis-MBP-tagged version of *Dm*Cdc6ΔN241 was purified in a similar manner except that TEV cleavage and the second nickel-affinity chromatography step were omitted. All purification steps were performed at 4 °C.

### Expression and purification of *Dm*Cdt1

*Drosophila* Cdt1 was expressed in BL21 RIL *E. coli* (cultured in Terrific Broth (TB)) upon induction with 0.5 mM Isopropyl β-D-1-thiogalactopyranoside (IPTG) overnight at 16 °C. Eight liters of culture were harvested, resuspended in 120 mL lysis buffer (50 mM Tris-HCl, pH 7.8, 1 M NaCl, 10% glycerol, 50 mM imidazole, pH 7.8, 5 mM BME, 200 μM PMSF, 1 μg/mL leupeptin) and lysed by sonication. The lysate was clarified by 30 min centrifugation at 30,996 × *g* in a Beckman Coulter Avanti J-26 XP centrifuge using a JA-17 rotor. Soluble Cdt1 in the supernatant was bound to a 5 mL HisTrap HP nickel-affinity chromatography column (GE Healthcare) and, after washing with 500 mL lysis buffer and 50 mL low salt buffer (50 mM Tris-HCl 7.8, 150 mM KCl, 10% glycerol, 50 mM imidazole, pH 7.8, 5 mM BME), eluted onto a 5 mL HiTrap Q HP anion exchange column (GE Healthcare) with 250 mM imidazole in 50 mM Tris-HCl, pH 7.8, 150 mM KCl, 10% glycerol, 5 mM BME. The flow-through was pooled and digested with 6xHis-tagged TEV protease while being dialyzed into 50 mM Tris-HCl, pH 7.8, 300 mM KCl, 10% glycerol, 5 mM BME overnight. TEV protease and uncleaved Cdt1 were removed nickel-affinity chromatography. Cleaved Cdt1 was concentrated in a 30K Amicon Ultra-15 concentrator (Millipore) and further purified by gel filtration on a HiLoad 16/600 Superdex 200 pg column (GE Healthcare) equilibrated in 50 mM Tris-HCl, pH 7.8, 300 mM KCl, 10% glycerol, 1 mM DTT. Peak fractions containing *Dm*Cdt1 were pooled, concentrated, flash frozen, and stored at −80 °C. All purification steps were performed at 4 °C.

### Expression and purification of *Dm*Mcm2–7

Four liters of Hi5 cells were co-infected with high-titer baculoviruses encoding for *Drosophila* Mcm2–7 subunits for two days. Cells were harvested by centrifugation and resuspended in 130 mL lysis buffer (50 mM HEPES-KOH, pH 7.5, 200 mM potassium acetate, 10% glycerol, 50 mM imidazole, pH 7.5, 1 mM BME, 1 μg/mL leupeptin, 200 μM PMSF). After sonication, the lysate was clarified by two rounds of ultracentrifugation and ammonium sulfate precipitation as described for *Dm*ORC. Mcm2–7 were isolated using a 5 mL HisTrap HP nickel-affinity chromatography column (GE Healthcare) and eluted using a 50–250 mM imidazole gradient in 50 mM HEPES-KOH, pH 7.5, 200 mM potassium acetate, 10% glycerol, 1 mM BME. Eluted proteins were loaded onto an 8 mL amylose column (New England Biolabs), washed in 50 mM HEPES-KOH, pH 7.5, 200 mM potassium acetate, 10% glycerol, 1 mM BME, and Mcm2–7 was eluted by adding 20 mM maltose. Affinity tags were removed by overnight digestion with 6xHis-tagged TEV protease, followed by nickel-affinity and gel filtration chromatography using a Superose 6 Increase 10/300 GL column (GE Healthcare) in 25 mM HEPES-KOH, pH 7.5, 300 mM potassium glutamate, 10% glycerol, 1 mM DTT. *Dm*Mcm2–7 peak fractions were pooled, concentrated in a 30K Amicon Ultra-15 concentrator (Millipore), flash frozen and stored at −80 °C. All purification steps were performed at 4 °C. The presence of all six *Dm*Mcm2–7 subunits was verified SDS-PAGE and mass spectrometry, and the integrity of the hexameric complex was validated by negative-stain electron microscopy (4 μL of 80 nM Mcm2–7 were applied to continuous carbon EM grids and stained with 2% uranyl acetate), showing the expected assembly of subunits into open, hexameric rings (Fig. 1c).

### Assembly and purification of complexes for cryo-EM

Attempts to reconstitute *Drosophila* ORC·DNA·Cdc6 and ORC·DNA complexes with full-length proteins at the micromolar concentrations necessary for cryo-EM studies using open hole cryo-EM grids resulted in visible sample opaqueness when mixing ORC and Cdc6 with DNA, and substantial protein aggregation was visible on cryo-EM grids. Removal of the N-terminal 439 amino acid residues of *Dm*Orc1 (referred to as Orc1ΔN) and the N-terminal 241 amino acid residues of *Dm*Cdc6 (Cdc6ΔN), which have been reported to drive liquid-liquid phase separation^32^, improved the behavior of these proteins in the presence of DNA without impeding *Dm*ORC’s ATPase activity, the ATP-dependent co-association of ORC, DNA, and Cdc6, and Mcm2–7 loading (Fig. 1, Supplementary Fig. 1). Therefore, these trimmed but Mcm2–7 loading-competent constructs were used to assemble *Dm*ORC·DNA and *Dm*ORC·DNA·Cdc6 complexes for cryo-EM experiments.

To reconstitute *Drosophila* ORC·DNA·Cdc6 assemblies, *Dm*ORC^Orc1ΔN^, duplex DNA (60 bp AT-rich, 84 bp AT-rich, 60 bp poly(dA·dT), or 60 bp GC-rich; Supplementary Table 2), and *Dm*Cdc6ΔN were combined at a final concentration of 2.5 μM, 3 μM, and 6.8 μM, respectively, in buffer containing 25 mM HEPES-KOH, pH 7.6, 250 mM potassium acetate, 10 mM magnesium acetate, 1 mM DTT, and 1 mM ATP. Assembly reactions were incubated for 5 min at 25 °C after mixing ORC with DNA, and again after adding Cdc6. Subsequently, 300 μL of the reconstitution reaction were loaded onto a Superose 6 Increase 10/300GL column (GE Healthcare) equilibrated in 25 mM HEPES-KOH, pH 7.6, 250 mM potassium acetate, 10 mM magnesium acetate, 1 mM DTT, and 0.2 mM ATP to purify the ternary *Dm*ORC·DNA·Cdc6 complex by gel filtration chromatography. Peak fractions containing DNA (judged by absorbance at 254 nm), *Dm*ORC^Orc1ΔN^, and *Dm*Cdc6ΔN were pooled and concentrated in a 30K Amicon Ultra-4 concentrator (Millipore) to an absorbance of 0.7–1.4 at 280 nm for cryo-EM grid preparation. The ORC·DNA complex was assembled and purified in an analogous manner with the exception that Cdc6 was omitted in the reconstitution reaction.

### Cryo-EM data collection and image processing

3.5 μL of purified, concentrated ternary *Dm*ORC·DNA·Cdc6 or the binary *Dm*ORC·DNA complexes were applied to plasma-cleaned (using a 18.9%/81.1% H_2_/O_2_ gas mixture for 30 s at 5 W) 300-mesh R1.2/1.3 UltrAuFoil grids (Quantifoil Micro Tools GmbH), adsorbed for 10 s, and then frozen in liquid ethane using a Vitrobot Mark IV plunge freezer (Thermo Fisher Scientific). Cryo-EM grids were imaged in a Titan Krios transmission electron microscope (Thermo Fisher Scientific) operated at an acceleration voltage of 300 kV and equipped with a spherical aberration (Cs) corrector (CEOS GmbH), a Quantum LS energy filter, and a post-GIF Gatan K2 summit direct electron detector. Dose-fractionated images were collected in an automated manner with EPU (Thermo Fisher Scientific) in EFTEM mode (slit width 20 eV) with or without a Volta phase plate using a target defocus of 0.4–0.6 μm (with phase plate, aiming for a phase shift of 20–130 degrees) and 1–2.2 μm (without phase plate), respectively. For each dataset, 40 (with Volta phase plate) or 50 (without Volta phase plate) frames were recorded per movie at a dose rate of 5–7 e^−^/Å^2^ per second, yielding a total electron dose of 40 e^−^/Å^2^ and 50 e^−^/Å^2^, respectively (see also Supplementary Table 1 for additional information on data collection settings). Data of the *Dm*ORC·DNA·Cdc6 complex assembled with poly(dA·dT) DNA was collected as described above with the exception that a Falcon 3EC direct electron detector (Thermo Fisher Scientific) in electron counting mode was used to record dose-fractionated movies at a target defocus of 0.8–1.5 μm. Movies were recorded as 50 frames over 41.47 s at a dose rate of 1.21 e^−^/Å^2^ per second, resulting in a total dose of 50.2 e^−^/Å^2^.

Recorded movie frames were motion-corrected using MotionCor2^60^, and contrast transfer function parameters, including phase shifts, were determined from averaged movie frames with GCTF^61^. Subsequently, averaged movie images and power spectra were manually inspected and micrographs containing aggregated protein, severe ice contamination, or having GCTF resolution estimates of worse than 4–6 Å were excluded from further processing. For the first dataset, the ternary *Dm*ORC·DNA·Cdc6 complex assembled on 60 bp AT-rich DNA (dataset 1), particles were initially automatically picked in a reference-free manner using GAUTOMATCH (developed by Kai Zhang, https://www.mrc-lmb.cam.ac.uk/kzhang/Gautomatch/) and subjected to 2D classification. Class averages representing different views of the ternary complex were selected, low-pass filtered to 30 Å, and served as templates for reference-based particle selection with GAUTOMATCH for all datasets. All further EM image processing steps, including particle extraction, 2D and 3D classification, 3D refinement, particle polishing, and postprocessing were performed using RELION 2.1 and RELION 3.0^62–64^. Picked particles were extracted using a box size of 300 × 300 pixels, normalized, and subjected to 2D and 3D classification. To remove damaged or falsely picked particles, we performed one to two rounds of 3D classification of the data into four to eight classes, which yielded better results than 2D classification in retaining particles corresponding to underrepresented views of the complex. A low-resolution 3D reconstruction (low-pass filtered to 50 Å) from negatively stained particles of the ternary complex served as initial reference for 3D classification of the *Dm*ORC·DNA·Cdc6 dataset 1. For all other datasets, the cryo-EM volume of the ternary complex, low-pass filtered to 50 Å, provided the starting reference for 3D classification. Particles of 3D class volumes with clearly visible secondary structure elements (cleaned particles) were subjected to 3D autorefinement (with a soft-edged, global mask low-pass filtered to 15 Å) and particle polishing. B-factors were automatically estimated in RELION 3.0 and used for map sharpening as implemented in the RELION postprocessing procedure. Global resolution of entire maps and local resolutions of masked sub-regions were determined using gold-standard Fourier shell correlations between half-maps in RELION 3.0.

### Model building and refinement

Model building was initiated by individually docking the AAA+ and winged-helix domains of the Orc1–5 subunits of the *Dm*ORC crystal structure (PDB 4xgc^16^) into the cryo-EM density map of *Dm*ORC·DNA·Cdc6 (with 60 bp AT-rich dsDNA, dataset 1) using UCSF Chimera^65,66^. The *Dm*ORC model was then manually rebuilt in COOT^67^ to accommodate for structural changes in *Dm*ORC between the *apo-*ORC state and the ternary complex structure, and to build additional segments that were poorly or not resolved in the *Dm*ORC crystal structure. For model building, a combination of unsharpened, globally sharpened, and locally sharpened (with LocScale^68^) cryo-EM density maps were used. *Dm*Cdc6 was built de novo in COOT into the sharpened EM density, which was of sufficient resolution to almost completely trace the main chain and to assign the amino acid register to the density. The close evolutionary kinship between *Dm*Cdc6 and *Dm*Orc1 provided additional information on the topological fold of *Dm*Cdc6 to confirm our assignment. For initial DNA placement, short segments of idealized B-form DNA were docked into the cryo-EM map using UCSF Chimera and locally rebuilt in COOT. The resulting *Dm*ORC·DNA·Cdc6 model was improved by iterative rounds of real-space refinement against the sharpened cryo-EM map in PHENIX^69,70^ using secondary structure restraints for both protein and DNA, as well Ramachandran and rotamer restraints for protein chains, and subsequent rebuilding in COOT. In addition, a morphing step was included during the first refinement round in Phenix. The final model has excellent geometry (MolProbity score 1.52^71^; Supplementary Table 1) and includes the AAA+ and winged-helix domains of Orc1–5 and Cdc6, the basic patch region in Orc1, the C-terminal helix in Orc6, and 34 base pair duplex DNA bound by *Dm*ORC·Cdc6.

Models for all other *Dm*ORC·DNA·Cdc6 assemblies, for the binary *Dm*ORC·DNA complex, and for DNA-free *Dm*ORC were built using the ternary complex with the 60 bp AT-rich duplex as a starting model. Briefly, the pdb model was docked into the respective cryo-EM density in UCSF Chimera, locally rebuilt in COOT if necessary, and real-space-refined in PHENIX using a combination of rigid body, XYZ, and ADP refinement. For cryo-EM maps of a resolution worse than 3.5 Å, we refrained from extensive rebuilding the corresponding coordinate models in COOT. We note that the assignment of DNA sequences in our models (except in those containing poly(dA·dT) duplexes) is speculative due to the sequence-independent DNA binding activity of *Dm*ORC. Consequently, individual particles in the 3D reconstructions likely bind DNA in different registers, which is consistent with the higher model and map B-factors in this region of the complex. Moreover, although full-length *Dm*Orc6 was used to reconstitute the binary and ternary complexes, no density was visible for the Orc6 TFIIB domain in 2D class averages or any of the 3D reconstructions, indicating flexibility of this region.

### Structure analysis

Structural alignments and superpositions were performed with PyMOL (The PyMOL Molecular Graphics System, Version 1.8.2.0 Schrödinger, LLC). Buried solvent accessible surface areas were determined using the PDBePISA server^72^ implemented in COOT. Protein–DNA contacts were explored using PDBePISA and DNAproDB^73^. Multiple protein sequence alignments were performed with MAFFT^74,75^ and visualized in JALVIEW^76^ or by generating sequence logos with WEBLOGO^77^. DNA shape parameters were determined using CURVES+^78,79^ and 3DNA^80,81^. Figures were rendered using PyMOL, UCSF Chimera, and UCSF ChimeraX^82^.

### Annealing of DNA duplexes

DNA duplexes used for reconstituting DNA-bound *Dm*ORC assemblies for structural studies and for biochemical assays were obtained by annealing single-stranded, complementary DNA oligonucleotides at 10 or 50 μM, respectively, each in 10 mM Tris-HCl, pH 8 and 5 mM MgCl_2_. For annealing, oligonucleotides were heated to 95 °C for 5 min in a 1 L water bath and slowly cooled to room temperature overnight. Successful annealing was verified by separating 0.25 pmol dsDNA on a 12% native-PAGE gel in 1× Tris-borate buffer pH 8.3 and subsequent staining with SYBR Safe (Thermo Fisher Scientific). DNA duplexes used throughout this study are summarized in Supplementary Table 2.

### Fluorescence anisotropy DNA binding assays

Binding of full-length wild type or mutant *Dm*ORC, or of truncated *Dm*ORC^Orc1ΔN^ and *Dm*ORC^Orc6ΔTFIIB^, to DNA was measured by fluorescence anisotropy using 40 bp dsDNAs (Supplementary Table 2) with one strand 5′-labeled with fluorescein similarly as done previously^18^. 1 nM dsDNA was mixed with increasing concentrations of *Dm*ORC (from 0.04 nM to 2.5 μM) in 25 mM HEPES-KOH, pH 7.6, 300 mM potassium glutamate, 10% glycerol, 10 mM magnesium acetate, 0.1% NP40, 1 mM DTT, 0.1 mg/mL BSA, and 0 or 1 mM ATP. After 40 min incubation at 25 °C, fluorescence anisotropy was measured using excitation and emission filters of 485 nm and 520 nm, respectively, in a PHERAstar FSX plate reader (BMG Labtech). To obtain apparent dissociation constant (*K*_d, app_) for *Dm*ORC binding to the respective DNA duplexes, the means and standard deviation (SD) of data points of n independent experiments (as listed in each figure) were plotted and fit to the Hill equation using the GraphPad Prism software:

1 $$A = A_{\mathrm{f}} + \frac{{\left( {A_{\mathrm{b}} - A_{\mathrm{f}}} \right) * [{\mathrm{R}}]^h}}{{(K_{{\mathrm{d}},{\mathrm{app}}}^h + [{\mathrm{R}}]^h)}}$$

where *A*_f_ and *A*_b_ are the measured anisotropy of free and bound, fluorescently labeled DNA duplex, respectively, [R] is the concentration of *Dm*ORC, *K*_d, app_ is the apparent binding constant, and h is the Hill coefficient. For the poly(dA·dT) duplex, we additionally fit the binding data points to the quadratic binding equation to account for potential *Dm*ORC depletion at low concentrations due to its high affinity for this DNA:

2 $$A = A_{\mathrm{f}} + (A_{\mathrm{b}} - A_{\mathrm{f}}) * \frac{{[{\mathrm{L}}] + K_{{\mathrm{d}},{\mathrm{app}}} + [{\mathrm{R}}] - \sqrt {([{\mathrm{L}}] + K_{{\mathrm{d}},{\mathrm{app}}} + [{\mathrm{R}}])^2 - 4 * \left[ {\mathrm{L}} \right] * [{\mathrm{R}}]} }}{{2 * [{\mathrm{L}}]}}$$

where *A*_f_ and *A*_b_ are the measured anisotropy of free and bound, fluorescently labeled poly(dA·dT) duplex, respectively, [L] is the concentration of poly(dA·dT) used in binding assays, [R] is the concentration of *Dm*ORC, and *K*_d, app_ is the apparent binding constant. *K*_d, app_ values obtained for both methods were with 2.1 nM (Hill equation) and 1.7 nM (quadratic equation) comparable. For simplicity, only Hill curves are shown in Supplementary Fig. 10a–c. For bar graphs, *K*_d, app_ and standard error (SE) of fits are plotted. DNA binding of *Dm*ORC protein in the presence of *Dm*Cdc6 was measured as described above with the exception that wild type or truncated *Dm*Cdc6 was added at equimolar concentrations to *Dm*ORC in the reaction mix.

### Pull-down assays

To verify that N-terminal truncations of *Dm*Orc1 and *Dm*Cdc6 do not interfere with ternary complex formation, we performed pull-down assays using MBP-tagged *Dm*Cdc6 or 6xHis-MBP-tagged *Dm*Cdc6ΔN as baits. 400 μL reactions were assembled with 60 bp AT-rich dsDNA (Supplementary Table 2) and different combinations of full-length *Dm*ORC or *Dm*ORC^Orc1ΔN^, and full-length MBP-*Dm*Cdc6 or 6xHis-MBP-*Dm*Cdc6ΔN at final concentrations of 100 nM, 100 nM, and 200 nM, respectively, in binding buffer containing 25 mM HEPES-KOH, pH 7.6, 300 mM potassium glutamate, 10 mM magnesium acetate, 10% glycerol, 1 mM DTT, and 0 or 1 mM ATP. *Dm*ORC and DNA were incubated for 15 min at 25 °C prior to the addition of *Dm*Cdc6. After an additional 15 min at 25 °C, reactions were added to 25 μL amylose beads (80% slurry, New England Biolabs) and incubated for 30 min at 25 °C. Beads were then washed twice with 1 mL binding buffer, followed by elution of bound proteins in 25 μL binding buffer supplemented with 20 mM maltose. 0.5% input and 32% eluted proteins were analyzed by 10% SDS-PAGE and silver staining. Pull-down assays were performed three times as independent experiments.

### ATPase activity assays

Steady-state ATP hydrolysis activity of *Dm*ORC was measured using the enzymatic NADH-coupled assay^83^. In this system, ATP hydrolyzed by *Dm*ORC is regenerated by pyruvate kinase, a reaction that is in turn coupled to the lactate dehydrogenase-mediated oxidation of NADH to NAD^+^, which is measured spectrophotometrically. Full-length wild type or mutant *Dm*ORC, or *Dm*ORC^Orc1ΔN^, were diluted to 2 μM and mixed with 2.4–4 units/mL pyruvate kinase/3.6–5.6 units/mL lactate dehydrogenase (PK/LDH from rabbit muscle, Sigma Aldrich), 8 mM phosphoenolpyruvate (Sigma Aldrich), 0.6 mM NADH (Sigma Aldrich) in buffer containing 25 mM HEPES-KOH, pH 7.6, 300 mM potassium glutamate, 10 mM magnesium acetate, 10% glycerol, 0.2% NP40, 2 mM DTT, 0.2 mg/mL BSA. 25 μL of this 2x *Dm*ORC/PK/LDH mix were transferred to a 96-well, half area microplate (Greiner) and reactions were initiated by adding 25 μL of 2x-ATP titrations (in two-fold steps, either in the absence or presence of 4 μM DNA duplex) in 25 mM HEPES-KOH pH 7.6, 300 mM potassium glutamate, 10% glycerol, 10 mM magnesium acetate. Final reactions (50 μL) therefore contained 1 μM *Dm*ORC assemblies, 1.2–2 units/mL PK/1.8–2.8 units/mL LDH, 4 mM phosphoenolpyruvate, and 0.3 mM NADH in 25 mM HEPES-KOH, pH 7.6, 300 mM potassium glutamate, 10% glycerol, 10 mM magnesium acetate, 0.1% NP40, 1 mM DTT, 0.1 mg/mL BSA. Final ATP concentrations in titration reactions ranged from 4 mM to 2 μM, serially 2-fold diluted. For DNA inhibition experiments, the 60 bp AT-rich DNA duplex was included in reactions at a final concentration of 2 μM. The decrease in NADH concentration resulting from ATP hydrolysis was monitored by measuring absorbance at 340 nm in a PHERAstar FSX plate reader (BMG Labtech) at 27 °C for 2 h at 1 min intervals. Raw absorbance values were converted to NADH concentrations using a standard curve of NADH titrations (in 2-fold steps, as well as 0 mM) in assay buffer conditions included on each 96-well plate measured. ATP hydrolysis rates were extracted for each ATP titration from the linear portions of the NADH consumption curves. The means and standard deviations of hydrolysis rates from three or more independent experiments were plotted as a function of ATP concentration and fit to the standard Michaelis–Menten equation using GraphPad Prism:

3 $$v = \frac{{V_{{\mathrm{max}}} * [{\mathrm{ATP}}]}}{{K_{\mathrm{M}} + [{\mathrm{ATP}}]}}$$

where *v* is the rate of ATP hydrolysis at a given substrate concentration (ATP), *V*_max_ is the maximum velocity of ATP hydrolysis, and *K*_M_ is the Michaelis–Menten constant. For bar graphs, *k*_cat_ and the standard error of the corresponding fits are plotted. Steady-state ATP hydrolysis activity of *Dm*Cdc6 and *Dm*Cd6ΔN were determined as described for *Dm*ORC. To investigate steady-state ATP hydrolysis of full-length and truncated ternary *Dm*ORC·DNA·*Dm*Cdc6 complexes, assays were set up in the presence of DNA as described above with the exception that 2 μM *Dm*Cdc6 or *Dm*Cd6ΔN was added to the 2x *Dm*ORC/PK/LDH mix.

### Mcm2–7 loading assay

In vitro Mcm2–7 loading assays using *Drosophila* proteins were adapted from those established previously for the *S. cerevisiae* system^46,84^. 0.6 pmoles of biotinylated duplex DNA (Supplementary Table 2) were coupled to 15 μL streptavidin sepharose high performance beads (GE Healthcare) in 20 μL coupling buffer (5 mM Tris-HCl, pH 8.0, 0.5 mM EDTA, 1 M NaCl, 0.1% NP40) for 30 min at room temperature. After washing with coupling buffer to remove free DNA, 0.6 pmoles streptavidin (Sigma Aldrich) were added for 15 min to block any free DNA ends and prevent Mcm2–7 sliding off after loading. Unbound streptavidin was removed and beads equilibrated by washing beads thrice in low salt buffer (25 mM HEPES-KOH, pH 7.6, 300 mM potassium glutamate, 10 mM magnesium acetate, 10% glycerol, 0.1% NP40, 1 mM DTT) in the absence or presence of 1 mM ATP. *Dm*ORC, *Dm*Cdc6, *Dm*Cdt1, and *Dm*Mcm2–7 were mixed at 250 nM, 250 nM, 500 nM, and 500 nM final concentration in low salt buffer with or without 1 mM ATP. 40 μL of the loading protein mix were added to DNA-coupled beads and incubated for 30 min at 27 °C. To assess Mcm2–7 loading, beads were washed once with high salt buffer (25 mM HEPES-KOH, pH 7.6, 1 M KCl, 10 mM magnesium acetate, 10% glycerol, 0.1% NP40, 1 mM DTT, 0 or 1 mM ATP), followed by two washes with low salt buffer (25 mM HEPES-KOH, pH 7.6, 300 mM potassium glutamate, 10 mM magnesium acetate, 10% glycerol, 0.1% NP40, 1 mM DTT, 0 or 1 mM ATP). To analyze Mcm2–7 recruitment, beads were washed with low salt buffer instead of high salt buffer. Bound proteins were eluted by digestion with 1000 units micrococcal nuclease (New England Biolabs) for 10 min at 37 °C in 25 mM HEPES-KOH, pH 7.6, 300 mM KCl, 10 mM magnesium acetate, 10% glycerol, 0.1% NP40, 5 mM CaCl_2_, 1 mM DTT, 0 or 1 mM ATP, and analyzed by SDS-PAGE and silver staining. All loading assays were performed at least twice as independent experiments. 4 μL of eluted proteins were also applied to glow-discharged continuous carbon EM grids and stained with 2% uranyl formate prior to imaging in a FEI Tecnai T12 Spirit transmission electron microscope to verify double hexamer formation (Fig. 1c, Supplementary Fig. 1e).

### Reporting summary

Further information on research design is available in the Nature Research Reporting Summary linked to this article.

## Supplementary information

Supplementary Information

Peer Review File

Reporting Summary

Description of Additional Supplementary Files

Supplementary Movie 1

## Data Availability

The data that support this study is available from the corresponding author upon reasonable request. The cryo-EM maps and model coordinates have been deposited in the Electron Microscopy Databank (EMDB) and Protein Databank (PDB), respectively, under the following accession numbers: EMD-22361 and PDB 7JK4 (ORC·DNA·Cdc6, 60 bp AT-rich), EMD-22362 and PDB 7JK5 (ORC·DNA, 60 bp AT-rich), EMD-22363 and PDB 7JK6 (ORC, active conformation), EMD-22329 and PDB 7JGR (ORC·DNA·Cdc6, 84 bp AT-rich), EMD-22360 and PDB 7JK3 (ORC·DNA·Cdc6, 60 bp GC-rich), EMD-22359 and PDB 7JK2 (ORC·DNA·Cdc6, 60 bp poly(dA·dT) state 1), and EMD-22330 and PDB 7JGS (ORC·DNA·Cdc6, 60 bp poly(dA·dT) state 2). Plasmids generated in this study are available upon request from the corresponding author. Source data are provided with this paper.

## Source data

Source Data
