# Supplementary material for: Structural mechanism for replication origin binding and remodeling by a metazoan origin recognition complex and its co-loader Cdc6
Source: Nat Commun. 2020 Aug 26;11:4263. doi: 10.1038/s41467-020-18067-7 (PMC7450096; doi:10.1038/s41467-020-18067-7)
Supplement: Supplementary file 1 — Supplementary Information [file 41467_2020_18067_MOESM1_ESM.pdf]

Structural Mechanism for Replication Origin Binding and Remodeling by a Metazoan Origin Recognition Complex and its Co-loader Cdc6

Schmidt and Bleichert

Supplementary Information

Supplementary Figures 1-10

Supplementary Tables 1-2

Suplementary References

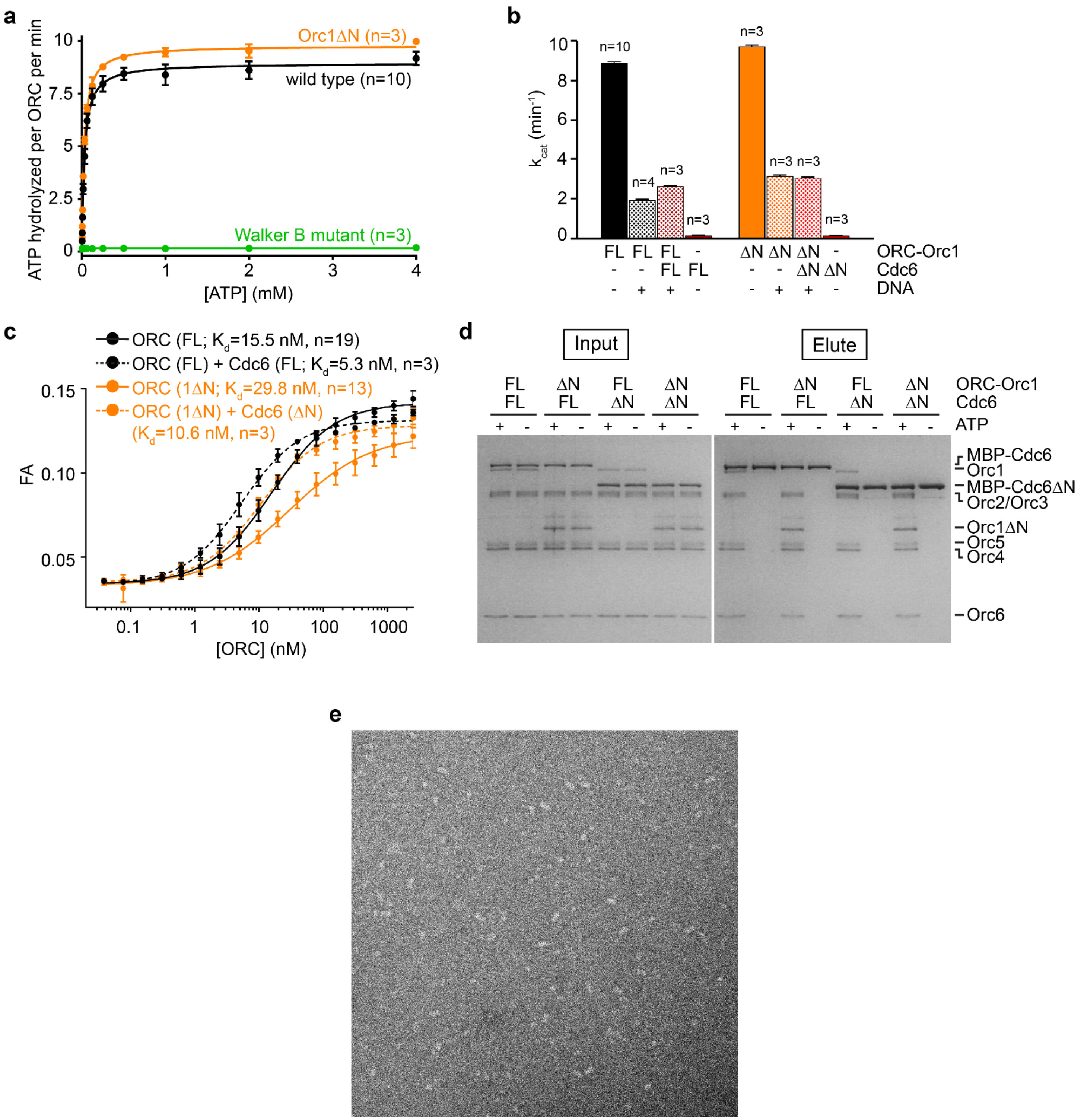

Supplementary Figure 1. **Reconstitution of an Mcm2-7 loading-competent, ternary *Dm*ORC· DNA·Cdc6 complex. a**) Full-length (wild type) *Dm*ORC and *Dm*ORC lacking the N-terminal 439 amino acids of Orc1 (Orc1ΔN) hydrolyze ATP with similar rates (~9-10 ATP hydrolyzed per min per ORC at saturating ATP concentrations) following Michaelis-Menten kinetics. *Dm*ORC bearing a mutation in the conserved Walker B motif of Orc1 (Orc1^{D684A}) is catalytically inactive. The means and standard deviations of ATP turnover rates of independent replicates (n is indicated) are plotted as a function of ATP concentration and fit to the Michaelis-Menten equation. **b**) ATPase activity of ORC is altered by DNA but not Cdc6. k_{cat} and S.E. of Michaelis-Menten equation fits to ATP titrations from independent experiments (n is indicated) are plotted. **c** and **d**) Removal of

the *Dm*Orc1 and *Dm*Cdc6 N-termini does not interfere with ternary complex formation. In **c**, ATP-dependent DNA binding by *Dm*ORC in the absence or presence of Cdc6 was measured by fluorescence anisotropy. Removal of the Orc1 N-terminal region has a <2-fold effect on *Dm*ORC's affinity for DNA, while addition of *Dm*Cdc6 or of N-terminally truncated *Dm*Cdc6 (*Dm*Cdc6ΔN) slightly stabilizes ORC on DNA (~3-fold decrease in $K_{d,\ app}$). Mean and standard deviations of n independent replicates (see figure for n) are plotted, and apparent dissociation constants ($K_{d,\ app}$) are listed. In **d**, Pull-down assays using MBP-tagged Cdc6 as bait show ATP-dependent co-purification of *Dm*ORC in the presence of 60 bp AT-rich dsDNA to similar extents when full-length (FL) or truncated (ΔN) Cdc6 or Orc1 (in the context of hexameric ORC) are used. Input (0.5%) and eluted proteins were separated by SDS-PAGE and visualized by silver staining. **e**) Negative-stain electron micrograph of loaded Mcm2-7. Source data are provided as a Source Data file.

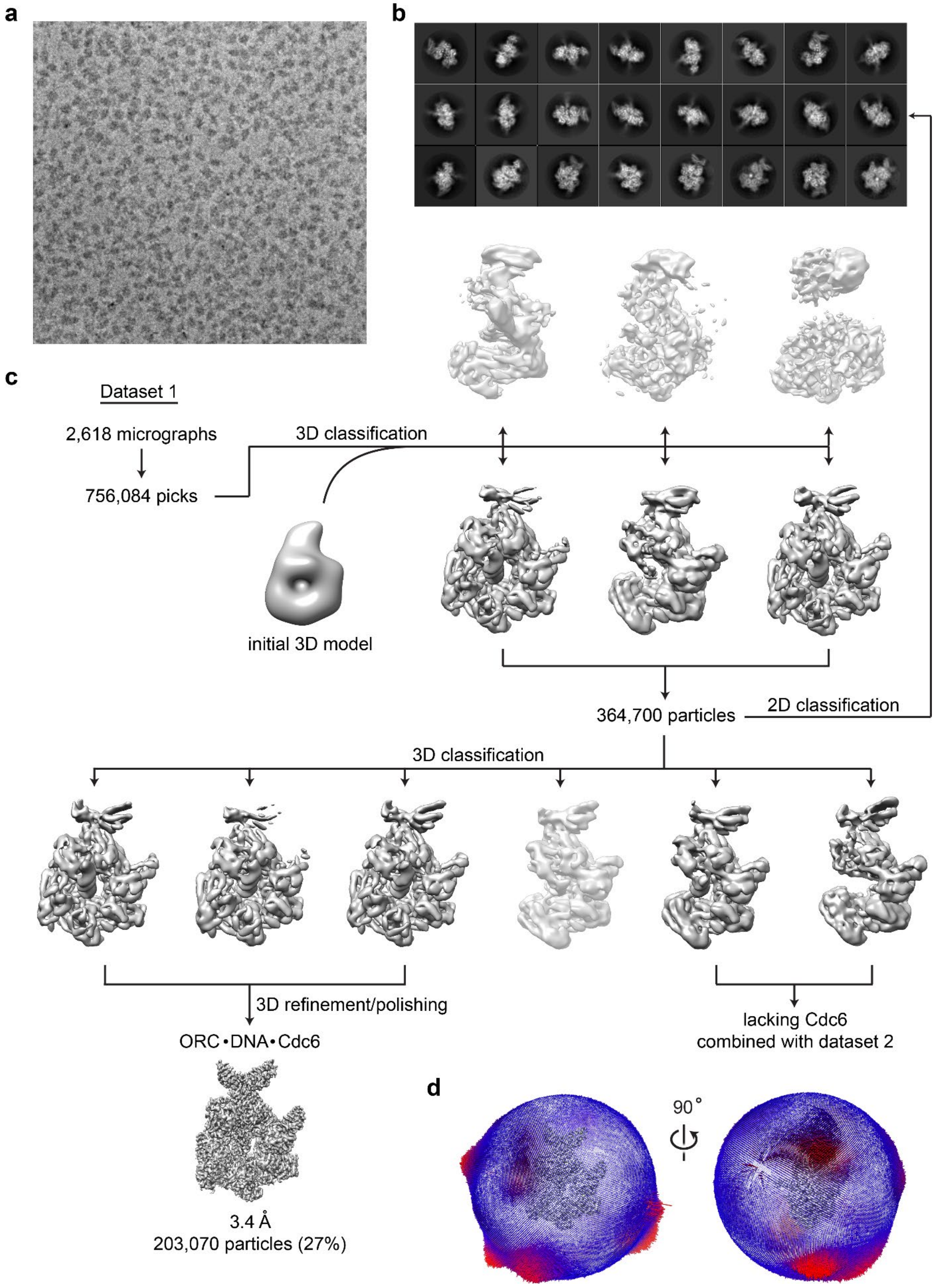

Supplementary Figure 2. **Cryo-EM data collection and processing workflow for the ternary *Dm*ORC·DNA·Cdc6 complex assembled on an AT-rich 60 bp duplex (dataset 1).** Representative **a**) electron micrograph and **b**) 2D class averages. **c**) Two rounds of 3D classification were performed to sort particles, and

those in well-resolved 3D classes (dark grey) corresponding to DNA-free *Dm*ORC, the binary *Dm*ORC·DNA assembly, and the ternary *Dm*ORC·DNA·Cdc6 complex were kept for further processing. Particles from other classes (light grey) were discarded. *Dm*ORC·DNA·Cdc6 particles were subjected to 3D refinement and polishing, yielding a 3D map with an overall resolution of 3.4 Å. *Dm*ORC and *Dm*ORC·DNA particles were combined prior to 3D refinement with respective classes from a second dataset that was collected using the binary *Dm*ORC·DNA complex (see also **Supplementary Fig. 6**). **d**) Angular distribution of particles contributing to the final 3D reconstruction of *Dm*ORC·DNA·Cdc6. Similar processing schemes as outlined here were used for all other datasets.

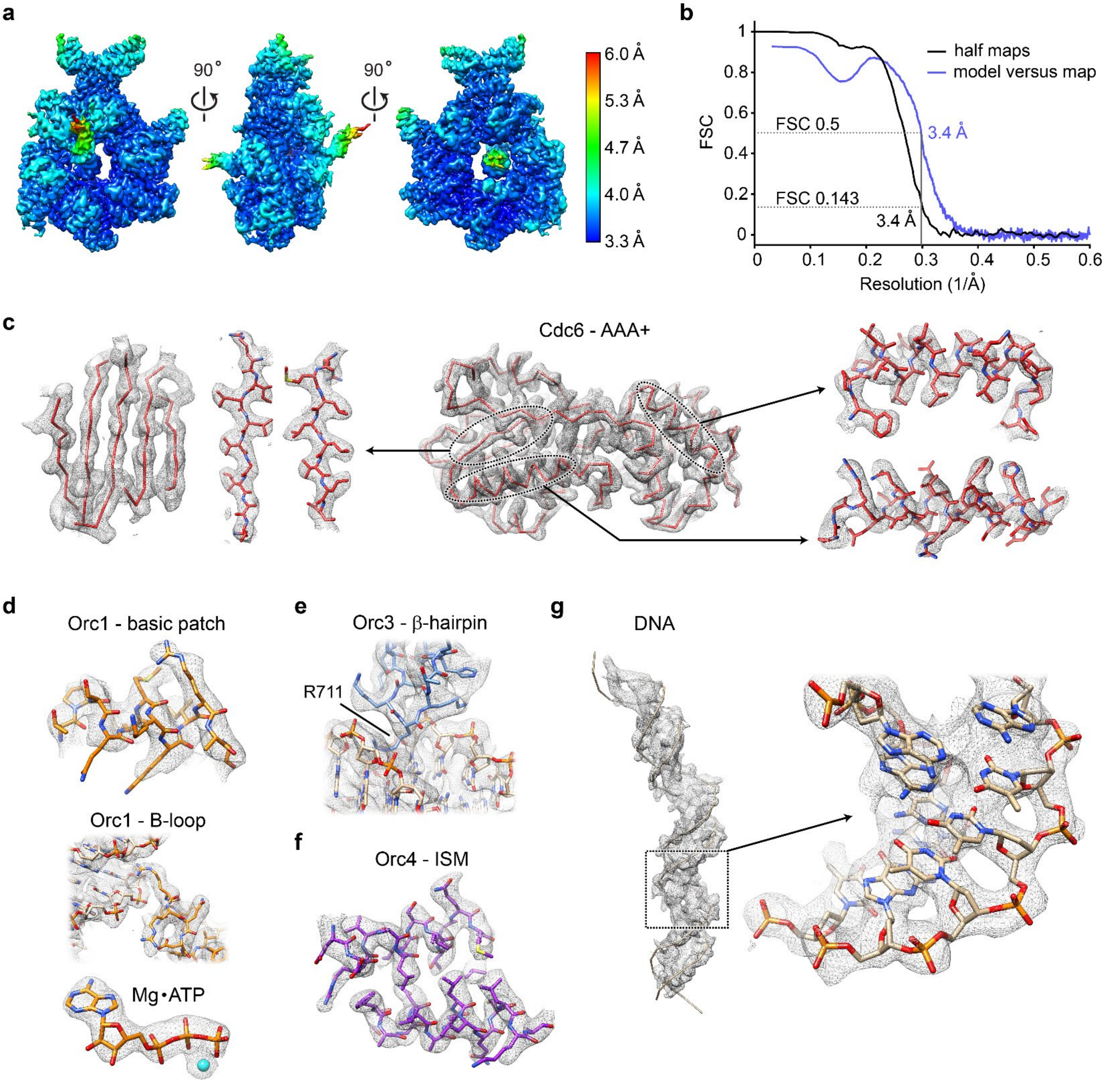

Supplementary Figure 3. **Local resolution, model building, and refinement of *Dm*ORC·DNA·Cdc6. a**) Unsharpened EM map of the ternary complex colored by local resolution. **b**) Fourier shell correlation (FSC) curves calculated using EM half-maps, or the full EM map and a pdb-derived model map. The resolutions at $FSC_{0.143}$ (for EM half-maps) and $FSC_{0.5}$ (for model versus EM map) are indicated. **c** to **g**) EM density map (sharpened and filtered to local resolution) and model is shown for various regions of the *Dm*ORC·DNA·Cdc6 structure.

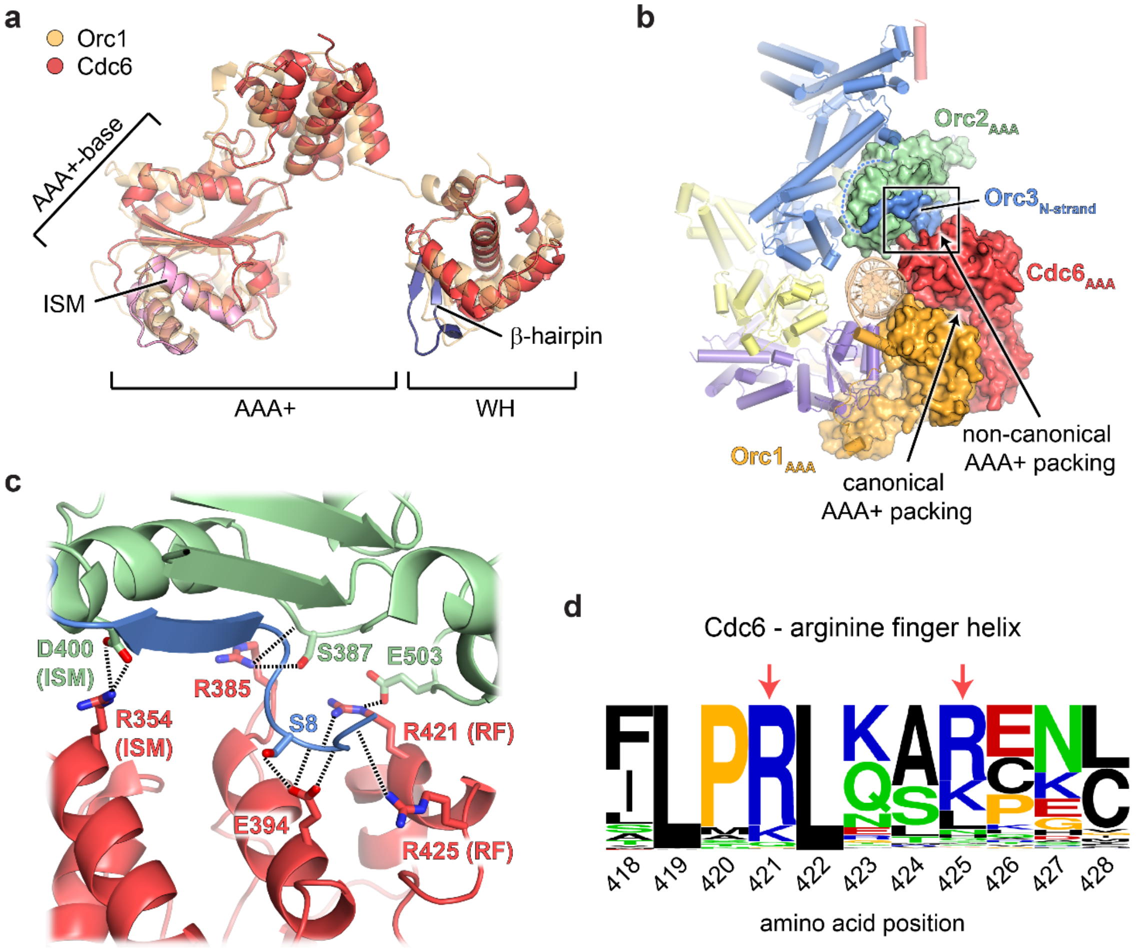

Supplementary Figure 4. **Molecular interactions between *Dm*Cdc6 and *Dm*ORC. a**) Superposition of Orc1 and Cdc6 by aligning their AAA+ base regions emphasizes the structural similarity of both proteins. The AAA+ initiator specific motif (ISM) and WH β-hairpin in Cdc6, two signature motifs in related archaeal and metazoan initiators, are colored pink and deep blue, respectively. **b**) *Dm*Cdc6 is recruited to *Dm*ORC by extensive, canonical AAA+/AAA+ interactions with Orc1, as well as by non-canonical AAA+/AAA+ packing with the Orc2 AAA+ domain and an additional β-strand in the Orc3 N-terminus. Modules involved in interactions are rendered as molecular surface, while other subunits and DNA are depicted as cartoon. The linker between the N-terminal Orc3 β-strand and the AAA+ core module is flexible (dashed line). **c**) Zoomed view of boxed region in **b** reveals numerous contacts between Cdc6 and the composite binding site formed by Orc2 and the Orc3 N-terminus. Residues in the ISM helix and arginine finger helix of Cdc6 contribute to the bonding network. **d**) Sequence frequency logo of a Cdc6 multiple sequence alignment illustrates the conservation of the arginines (red arrows) in the RF-helix that participate in interactions with Orc2 and Orc3. ISM – initiator specific motif, RF – arginine finger.

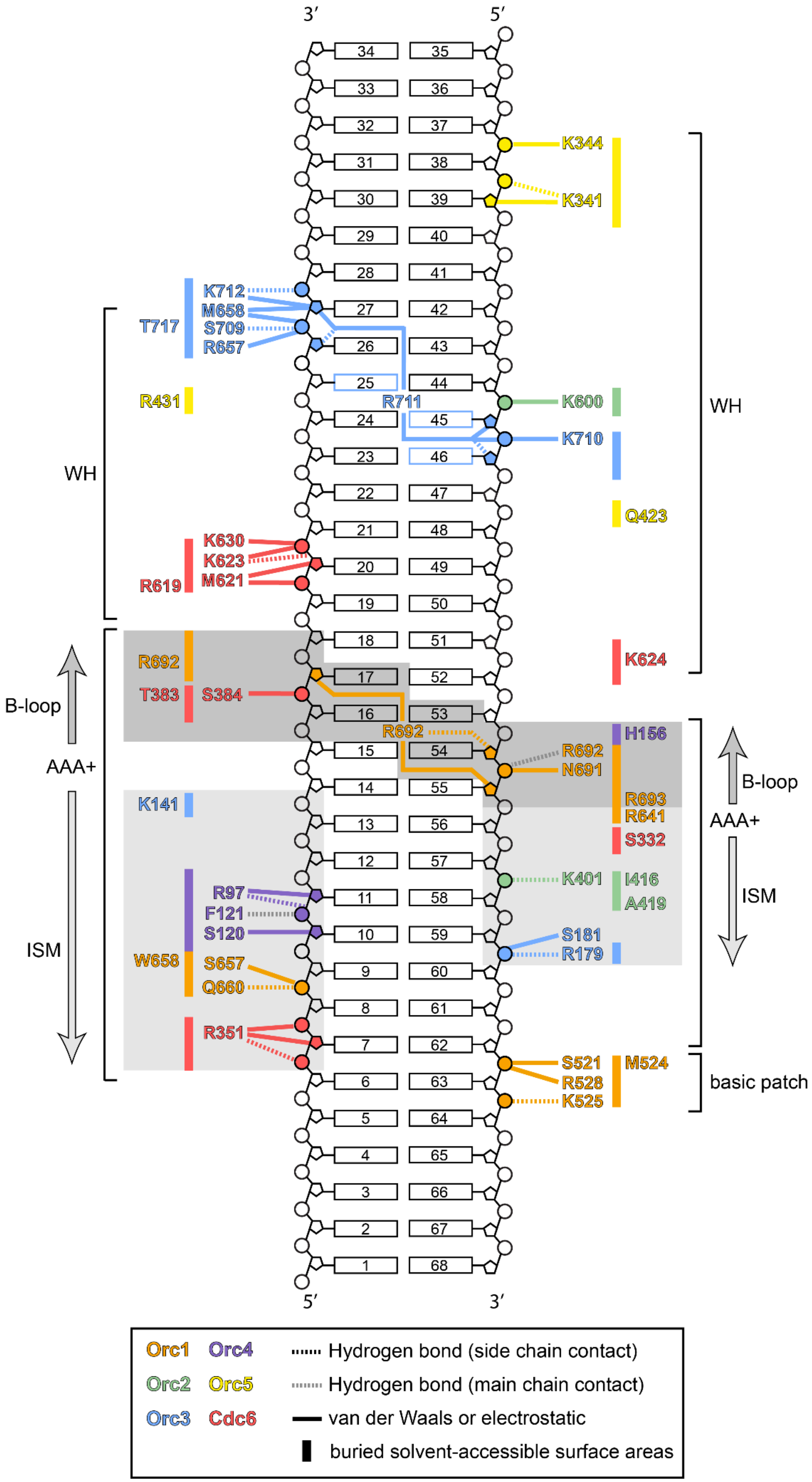
3′
5′
WH
B-loop
AAA+
ISM
basic patch
K344
K341
K712
M658
S709
R657
T717
R431
R711
K600
K710
Q423
K630
K623
M621
R619
K624
R692
T383
S384
R692
H156
R692
N691
R693
R641
S332
K141
K401
I416
A419
R97
F121
S120
S181
R179
W658
S657
Q660
R351
S521
R528
K525
M524
Orc1
Orc4
Orc2
Orc5
Orc3
Cdc6
Hydrogen bond (side chain contact)
Hydrogen bond (main chain contact)
van der Waals or electrostatic
buried solvent-accessible surface areas

Supplementary Figure 5. **Summary of DNA contacts by *Dm*ORC and Cdc6.** Residue contact map for the *Dm*ORC·DNA·Cdc6 complex (reconstituted with the 60 bp AT-rich DNA duplex). Amino acid residues within hydrogen bonding distance of DNA (3.6 Å cut-off), and those engaged in van der Waals or electrostatic interactions, are indicated by dashed and solid lines, respectively. Colored bars represent buried solvent-accessible surface areas. Interactions involving ISM and B-loop residues are highlighted by light grey and dark grey boxes.

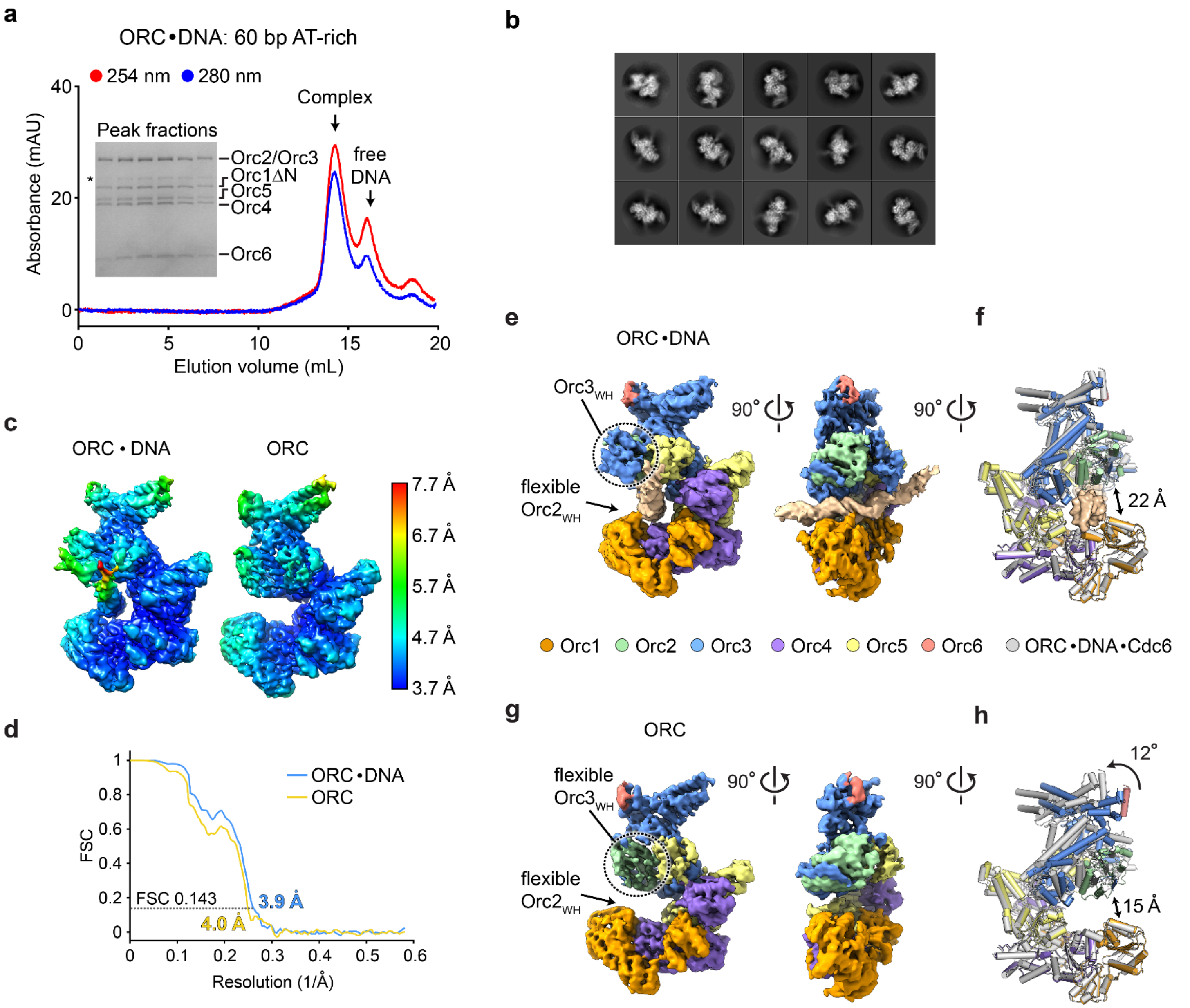

Supplementary Figure 6. **Cryo-EM structures of the binary *Dm*ORC·DNA complex and DNA-free *Dm*ORC. a**) Reconstitution and purification of *Dm*ORC·DNA. Gel filtration chromatogram and SDS-PAGE gel of complex peak fractions are shown. **b**) Representative 2D class averages obtained for the *Dm*ORC·DNA sample. Classification revealed that the sample contained a mixture of DNA-free and DNA-bound *Dm*ORC. **c**) Unsharpened cryo-EM maps for *Dm*ORC·DNA and DNA-free *Dm*ORC (ATP-bound) colored by local resolution. For 3D refinement of the final maps, DNA-bound and DNA-free *Dm*ORC 3D classes from dataset 2 were merged with the corresponding ones from dataset 1 (see **Supplementary Fig. 2**). Both EM volumes show an active ORC state with a large gap in the ORC ring that is otherwise occupied by Cdc6 in *Dm*ORC·DNA·Cdc6. **d**) Gold-standard FSC curves for *Dm*ORC and *Dm*ORC·DNA calculated using EM half-maps. The resolutions at $FSC_{0.143}$ are 3.9 Å and 4 Å (albeit slightly anisotropic due to particle orientation bias). **e** and **f**) Cdc6 binds *Dm*ORC·DNA by a lock-and-key-like mechanism and does not substantially remodel ORC·DNA contacts (except for Orc2 WH). **e**) WH and side views of the *Dm*ORC·DNA cryo-EM map (unsharpened) colored by subunit. No cryo-EM density is observed for the Orc2 WH domain which is detached from the AAA+ layer and flexible in the absence of Cdc6. **f**) Superposition of the *Dm*ORC model in the binary (cartoon, colored by subunit) and ternary (grey cartoon) complex. Cdc6 binding does not extensively remodel

the Orc1-5 ring (apart from the Orc2 WH module). **g** and **h**) DNA binding by ORC stabilizes the WH domain of Orc3 and induces a slight opening of the ORC ring. **g**) WH and side views of the DNA-free *Dm*ORC cryo-EM map (unsharpened) colored by subunit. The Orc3 WH domain is flexible. **h**) Structural comparison of the DNA-free, active *Dm*ORC model (cartoon, colored by subunit) with *Dm*ORC in the ORC·DNA·Cdc6 complex (grey cartoon). DNA binding widens the Orc1-5 ring and the gap between Orc1 and Orc2, and is accompanied by a ~12° rotation of the Orc2/Orc3 module with respect to Orc1/Orc4/Orc5. Source data are provided as a Source Data file.

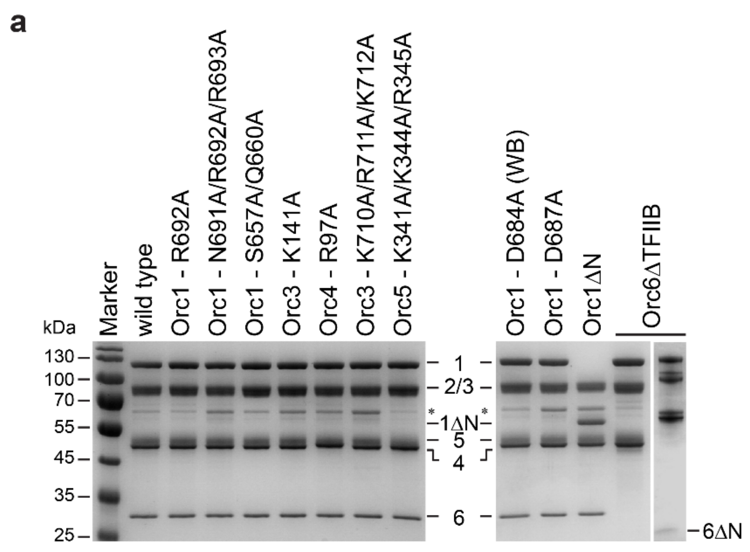

Supplementary Figure 7. **Purification and DNA binding analysis of mutant *Dm*ORC assemblies. a**) SDS-PAGE gel of purified wild type and mutant ORC assemblies used in this study. For Orc6ΔTFIIB, an additional higher percentage gel was run (right lane) to resolve the 8.1 kDa protein. The asterisk marks a degradation product of Orc2 and/or Orc3. **b**) *Dm*Cdc6 does not rescue the DNA binding defect resulting from mutations in the Orc1-B-loop. ATP-dependent DNA binding curves (determined by fluorescent anisotropy, means +/-SD of data points from independent replicates (n) are shown) for *Dm*ORC containing Orc1$^{N691A,\ R692A,\ R693A}$ in the absence and presence of *Dm*Cdc6. Source data are provided as a Source Data file.

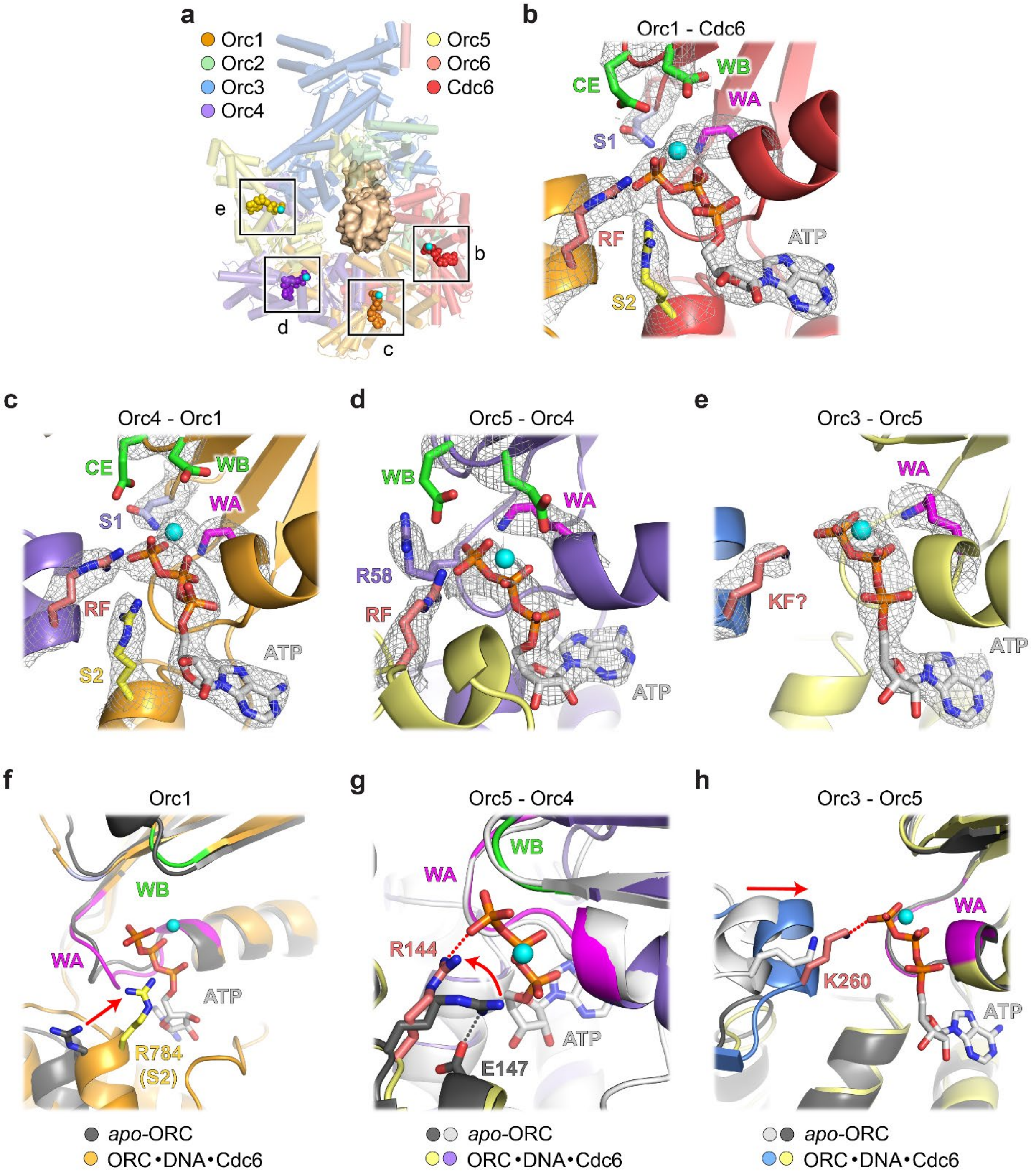

Supplementary Figure 8. **Configuration of ATP-binding centers in *Dm*ORC·DNA·Cdc6. a**) ATP (spheres, colored by subunit) and Mg^{2+} (cyan spheres) are bound at four AAA+/AAA+ interfaces: Cdc6/Orc1, Orc1/4, Orc4/5, and Orc5/3. **b** to **e**) Detailed view of each bipartite ATP-binding site with ATP and side chains of conserved active site residues (Walker A, Walker B, sensors 1 and 2, and arginine finger) shown as sticks. Compared to **a**, the ATPase sites are rotated to position the Walker B motif or corresponding regions at the top of each image. The grey mesh outlines the cryo-EM map density (sharpened) for ATP, Mg^{2+}, and displayed side chains. Note that only the Cdc6/Orc1 and Orc1/4 ATPase sites retain all active site residues and possess catalytic activity in yeast and metazoans. Consequently, both ATPase centers adopt a closed configuration

with conserved Walker A, sensor 1, sensor 2, and trans-acting arginine finger residues engaging the nucleotide triphosphate. The Walker B aspartate and catalytic glutamate side chains are not well resolved in the EM map. **f** to **h**) Comparison of ATPase centers in *Dm*ORC·DNA·Cdc6 and *apo-Dm*ORC crystallized previously (PDB 4xcg[1]). Except for the Orc1/4 ATPase center, which is not formed in *apo-Dm*ORC, the active sites are organized similarly in both structures with minor conformational changes. For example, the α-helical lid subdomain of Orc1 rotates towards the AAA+ base to position the conserved sensor 2 near the nucleotide (in **f**), while putative arginine and lysine fingers of Orc5 (in **g**) and Orc3 (in **h**) reorient or move into the ATP binding site to engage the γ-phosphate. These changes likely contribute to the ATP-mediated stabilization of hexameric ORC assemblies and/or the active ORC conformation[2-5]. Movements are highlighted by red arrows.

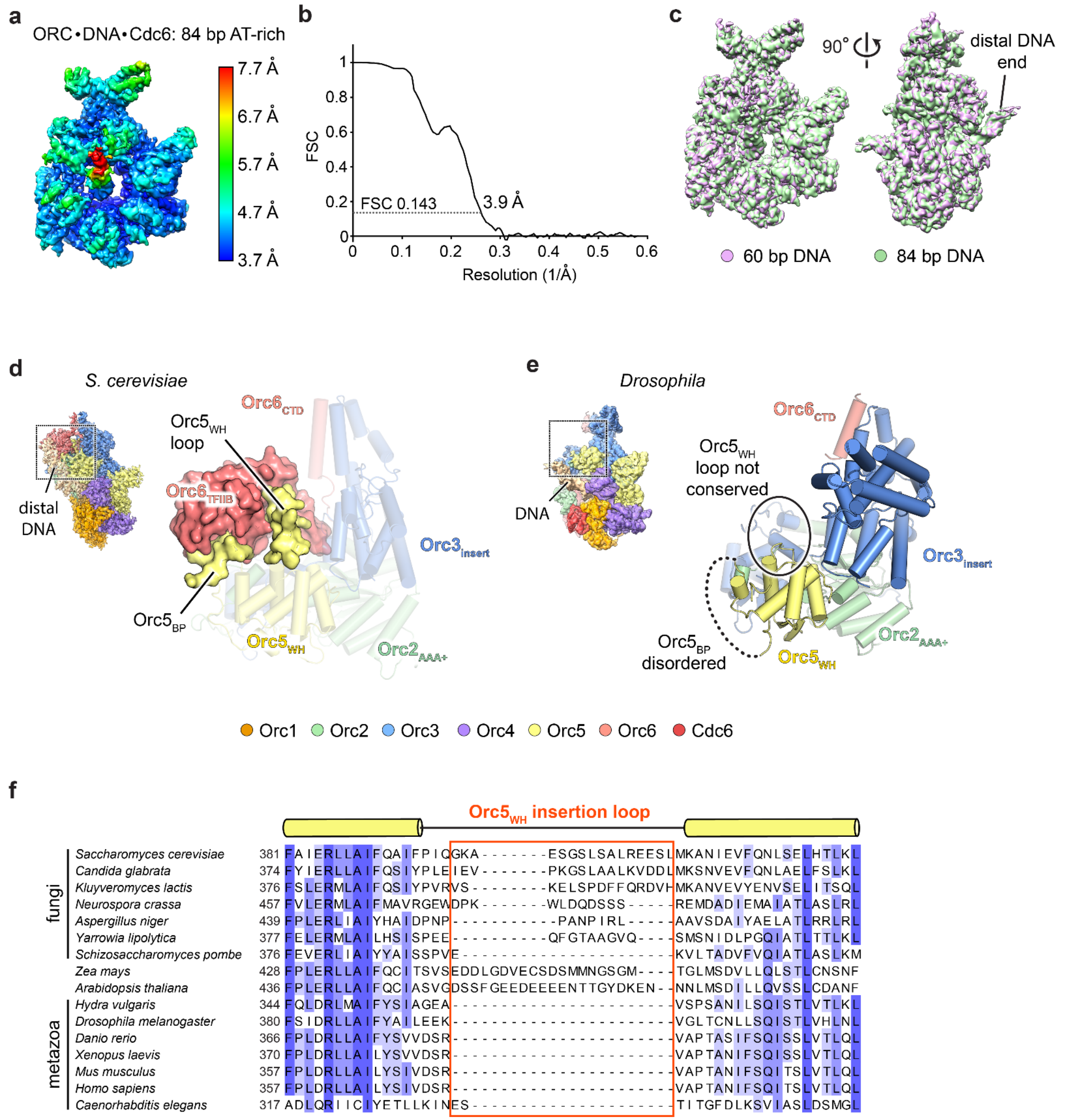

Supplementary Figure 9. Increasing duplex DNA length does not facilitate additional interactions between *Dm*ORC and DNA. **a**) Cryo-EM structure of a ternary *Dm*ORC·DNA·Cdc6 complex reconstituted using an 84 bp AT-rich duplex. The cryo-EM map (unsharpened) is colored by local resolution. **b**) Gold-standard FSC curve comparing EM half-maps of the structure in **a**. The overall map resolution is 3.9 Å at $FSC_{0.143}$. **c**) Cryo-EM maps (unsharpened) of *Dm*ORC·DNA·Cdc6 assembled on the 60 bp and 84 bp AT-rich duplexes are superposed. No additional DNA density is resolved distal of the DNA bend with the longer duplex. **d** and **e**) Orc6 contacts DNA and docks against the Orc5 WH domain in *S. cerevisiae* but not *Drosophila* ORC. **d**) In the cryo-EM density of *S. cerevisiae* ORC bound to 72 bp origin DNA[6] (left part of panel), the distal DNA

segment is well resolved and binds the TFIIB domain of Orc6. **e**) Contrariwise, the distal DNA segment is flexible and the Orc6 TFIIB domain is not seen in the EM density of *Drosophila* ORC·Cdc6 bound to an 84 bp duplex (compare boxed regions in **d** and **e**). Close-up views in **d** and **e** unveil that the Orc5-basic patch ($Orc5_{BP}$) and a nearby loop ($Orc5_{WH}$-loop), both of which facilitate docking of the Orc6 TFIIB domain in budding yeast ORC, are disordered ($Orc5_{BP}$) or absent ($Orc5_{WH}$-loop) in *Dm*Orc5. Orc6-TFIIB and Orc5 elements important for Orc6 docking are rendered as molecular surface in the zoomed view in **d**. **f**) Multiple sequence alignment of Orc5 protein sequences (colored by % identity) reveals that the $Orc5_{WH}$-loop is an insertion specific to fungi and is not observed in metazoan species.

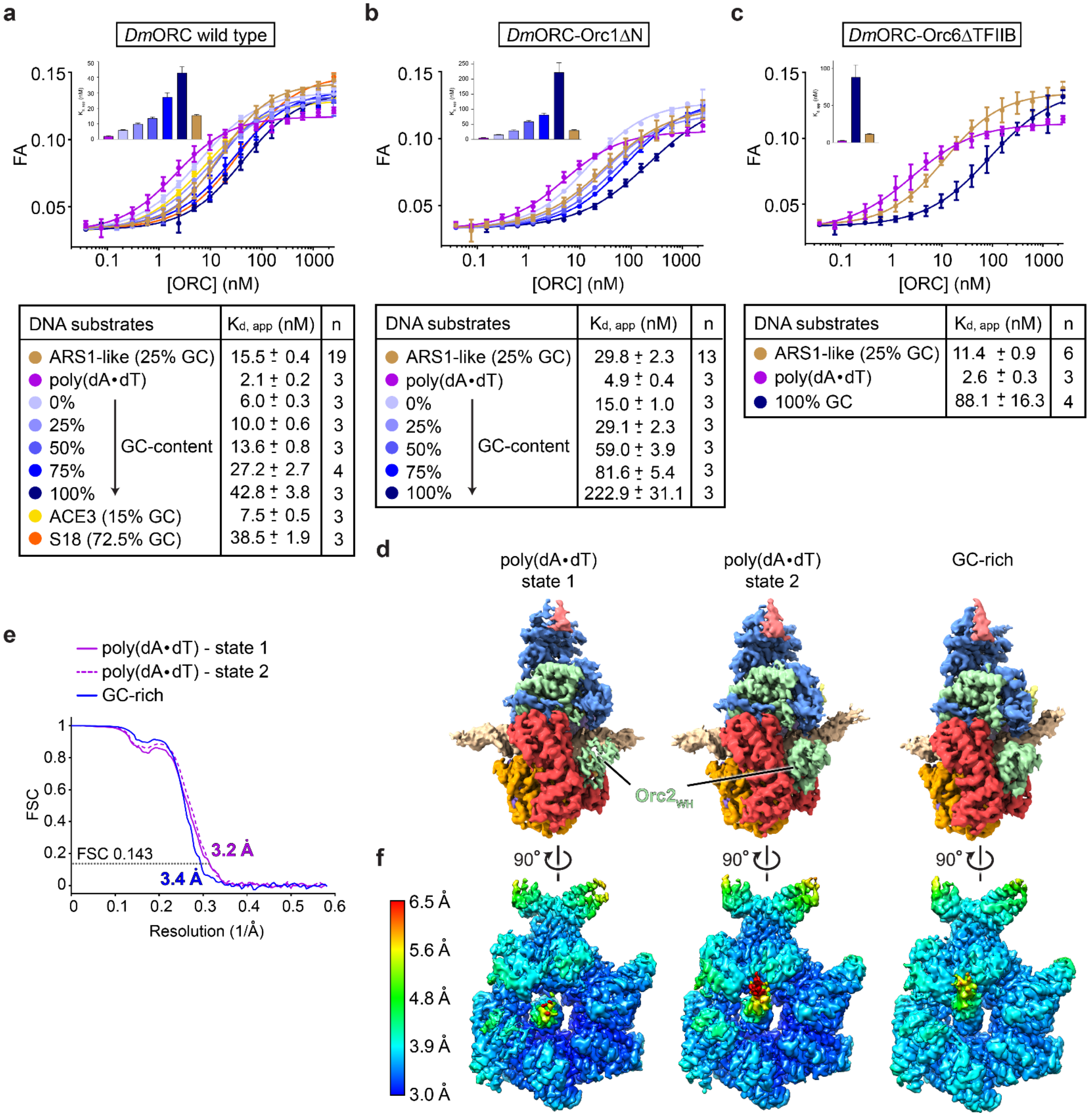

DNA substrates		$K_{d,\,app}$ (nM)	n
ARS1-like (25% GC)		15.5 ± 0.4	19
poly(dA•dT)		2.1 ± 0.2	3
0%	GC-content	6.0 ± 0.3	3
25%	GC-content	10.0 ± 0.6	3
50%	GC-content	13.6 ± 0.8	3
75%	GC-content	27.2 ± 2.7	4
100%	GC-content	42.8 ± 3.8	3
ACE3 (15% GC)		7.5 ± 0.5	3
S18 (72.5% GC)		38.5 ± 1.9	3

DNA substrates		$K_{d,\,app}$ (nM)	n
ARS1-like (25% GC)		29.8 ± 2.3	13
poly(dA•dT)		4.9 ± 0.4	3
0%	GC-content	15.0 ± 1.0	3
25%	GC-content	29.1 ± 2.3	3
50%	GC-content	59.0 ± 3.9	3
75%	GC-content	81.6 ± 5.4	3
100%	GC-content	222.9 ± 31.1	3

DNA substrates	$K_{d,\,app}$ (nM)	n
ARS1-like (25% GC)	11.4 ± 0.9	6
poly(dA•dT)	2.6 ± 0.3	3
100% GC	88.1 ± 16.3	4

Supplementary Figure 10. **DNA binding and remodeling of different DNA substrates by *Dm*ORC. a**) *Dm*ORC has a higher affinity for AT-rich DNA substrates than for GC-rich ones. DNA binding of wild type, full-length *Dm*ORC to fluorescein-labeled dsDNA was measured by fluorescence anisotropy. Mean and standard deviations of independent experiments (n is listed) are shown, and apparent dissociation constants ($K_{d,\,app}$) and standard errors of fits are summarized and also plotted as a bar graph in the inset. **b** and **c**) Removal of the N-terminal 439 amino acid residues in Orc1 (Orc1ΔN, in **b**) or the Orc6 TFIIB domain (Orc6ΔTFIIB, in **c**) does not mitigate *Dm*ORC's ability to associate more strongly with AT-rich duplexes as compared to GC-rich dsDNA. Fluorescence anisotropy experiments were performed as in **a**. **d** to **f**) Cryo-EM reconstructions of

*Dm*ORC·DNA·Cdc6 complexes reconstituted with a 60 bp poly(dA·dT) dsDNA and a 60 bp GC-rich duplex reveal different extents of DNA remodeling by *Dm*ORC. **d**) Side views of cryo-EM maps (unsharpened) of ternary complexes reconstituted with poly(dA·dT) and GC-rich dsDNA. In the case of the poly(dA·dT)-containing ternary complex, 3D classification revealed two distinct states characterized by different DNA bending angles. The DNA in state 1 exhibits a small DNA bend, while the degree of bending in state 2 is intermediate to state 1 and that observed for the GC- and AT-rich duplexes (see also **Figs. 6e-f**). The density for the Orc2 WH domain in state 1 is weak, suggesting that some DNA bending is necessary to allow docking of Orc2 WH onto the ORC·Cdc6 ring. **e**) Gold-standard FSC curves calculated using half-maps of each 3D reconstruction. The resolution at $FSC_{0.143}$ is 3.2 Å for both poly(dA·dT) states, and 3.4 Å for the reconstruction containing the GC-rich duplex. **f**) Unsharpened cryo-EM map for each reconstruction colored by local resolution. Source data are provided as a Source Data file.

Table 1. Summary of cryo-EM data collection, refinement, and validation statistics.

Sample	ORC·DNA·Cdc6 60 bp AT-rich (31.7% GC)	ORC·DNA 60 bp AT-rich (31.7% GC)	ORC·DNA·Cdc6 84 bp AT-rich (32.1% GC)	ORC·DNA·Cdc6 60 bp GC-rich (70% GC)		ORC·DNA·Cdc6 60 bp poly(dA·dT) (0% GC)
**EM data collection and processing:**						
	Dataset 1	Dataset 2	Dataset 3	Dataset 4	Dataset 5	Dataset 6
Microscope	Titan Krios (Volta phase plate, EFTEM)	Titan Krios (Volta phase plate, EFTEM)	Titan Krios (Volta phase plate, EFTEM)	Titan Krios (Volta phase plate, EFTEM)	Titan Krios (EFTEM)	Titan Krios
Camera	K2 Summit	K2 Summit	K2 Summit	K2 Summit	K2 Summit	Falcon 3EC (electron counting)
Voltage (kV)	300	300	300	300	300	300
Magnification	x130,000	x130,000	x130,000	x130,000	x130,000	x75,000
Frames (no.)	40	40	40	40	50	50
Total electron dose ($e^-/Å^2$)	40	40	40	40	50	50
Electron dose rate (e^-/pixel/s)	4.9	4.9	4.2	4.2	3.7	0.91
Calibrated pixel size (Å)	0.86	0.86	0.86	0.86	0.86	0.867
Defocus range (μm)	-0.4 to -0.6	-0.4 to -0.6	-0.4 to -0.6	-0.4 to -0.6	-1 to -2.2	-0.8 to -1.5
Micrographs	2,618	1,786	545	1,267	2,533	2,236
Initial picks (no.)	756,084	596,067	204,063	360,046	843,811	1,030,990

114,247

	ORC·DNA·Cdc6	ORC·DNA	ORC	ORC·DNA·Cdc6	ORC·DNA·Cdc6	ORC·DNA·Cdc6 state 1	ORC·DNA·Cdc6 state 2
Refined particles (no.)	203,070	131,750	126,560	44,520	174,891	82,964	81,248
Symmetry imposed	C1	C1	C1	C1	C1	C1	C1
Global resolution (Å)							
FSC 0.5 (unmasked/masked)	4.2/3.7	7.0/4.3	7.6/4.4	7.6/4.5	4.3/3.9	4.3/3.8	4.3/3.7
FSC 0.143 (unmasked/masked)	3.7/3.4	4.2/3.9	4.4/4.0	4.4/3.9	3.9/3.4	3.7/3.2	3.7/3.2
Local resolution range (Å)	3.2 - 7.5	3.7 - 8.5	3.7 - 7.0	3.7 - 11.3	3.3 - 6.4	3.0 - 9.6	3.0 - 11.1
Map sharpening B factor ($Å^2$)	-95	-138	-97	-94	-100	-82	-73
EMDB accession number	EMD-22361	EMD-22362	EMD-22363	EMD-22329	EMD-22360	EMD-22359	EMD-22330
**Model refinement and validation:**							
PDB accession number	7JK4	7JK5	7JK6	7JGR	7JK3	7JK2	7JGS
Model composition							
Non-hydrogen atoms	21717	17408	15161	21717	21661	21619	21743
Protein residues	2531	2003	1887	2531	2528	2523	2528
DNA residues	68	64	0	68	66	66	70
Ligands (ATP, Mg)	4, 4	3, 3	3, 3	4, 4	4, 4	4, 4	4, 4
Root mean square deviation							
Bond lengths (Å)	0.006	0.007	0.015	0.006	0.004	0.005	0.005
Bond angles (°)	0.878	1.044	1.207	0.878	0.784	0.846	0.875

	ORC·DNA·Cdc6 AT-rich	ORC·DNA AT-rich	ORC	ORC·DNA·Cdc6 AT-rich (84 bp)	ORC·DNA·Cdc6 GC-rich	ORC·DNA·Cdc6 poly(dA·dT) state 1	ORC·DNA·Cdc6 poly(dA·dT) state 2
B factors (Å^2)							
Protein	113.06	193.96	238.72	167.65	129.38	133.81	122.88
DNA	216.36	323.45	-	295.57	250.15	206.49	219.38
Ligands	92.2	152.81	195.84	140.86	105.63	101.69	96.42
Ramachandran plot							
% favored	95.89	95.1	95.6	95.89	96.85	96.48	97.3
% allowed	4.11	4.9	4.4	4.11	3.15	3.52	2.7
% outliers	0.0	0.0	0.0	0.0	0.0	0.0	0.0
Rotamer outliers (%)	0.32	0.29	0.24	0.36	0.18	0.23	0.18
MolProbity							
Clashscore	4.61	5.55	5.9	5.63	3.67	4.03	4.05
MolProbity score	1.52	1.64	1.63	1.59	1.35	1.42	1.32
Model-map comparison							
EM Ringer score*	3.11	1.52	0.69	1.84	2.28	2.71	2.92
CC_{mask}	0.88	0.84	0.78	0.84	0.87	0.86	0.87
$FSC_{model/map}$ 0.5	3.4	3.9	4.1	3.9	3.4	3.2	3.2

* The EM Ringer score was calculated using the B factor-sharpened cryo-EM maps.

Table 2. Summary of DNA oligonucleotides used in this study

Name	Sequence (5'-3')	used for
40 bp ARS1-like (25% GC)		DNA binding
Top	/5FluorT/TTTTGAAAAGCAAGCATAAAAGATCTAAACATAAAATCTG	
Bottom	CAGATTTTATGTTTAGATCTTTTATGCTTGCTTTTCAAAA	
40 bp poly(dA·dT)		DNA binding
Top	/5FluorT/TTTTTTTTTTTTTTTTTTTTTTTTTTTTTTTTTTTTTTTT	
Bottom	AAAAAAAAAAAAAAAAAAAAAAAAAAAAAAAAAAAAAAAA	
40 bp 0% GC		DNA binding
Top	/5FluorT/TATATTATATATATAAAATAAAATATATTATAAAATTTTT	
Bottom	AAAAATTTTATAATATATTTTATTTTATATATATAATATA	
40 bp 25% GC		DNA binding
Top	/5FluorT/TAGAATTTGAATGTATCCTTAGTCGATTCAAATCTAAATT	
Bottom	AATTTAGATTTGAATCGACTAAGGATACATTCAAATTCTA	
40 bp 50% GC		DNA binding
Top	/5FluorT/TGCCAGCTCTTTCAGTATCATGGAGCCCATGGTTGAGTGA	
Bottom	TCACTCAACCATGGGCTCCATGATACTGAAAGAGCTGGCA	
40 bp 75% GC		DNA binding
Top	/5FluorT/TCCCGGCGCCTGGGTGGTCGTACTGCCAGCTGAGCCCGCG	
Bottom	CGCGGGCTCAGCTGGCAGTACGACCACCCAGGCGCCGGGA	
40 bp 100% GC		DNA binding
Top	/5FluorT/CGCCGGGGCCGGGGGCGCCCCGCCGCGCGGGGCCGGGCCC	
Bottom	GGGCCCGGCCCCGCGCGGCGGGGCGCCCCCGGCCCCGGCG	
40 bp ACE3 (15% GC)		DNA binding
Top	/5FluorT/ GTTTATAATTTTATTGTAATTTTATCTCAATTTTTTTTGC	
Bottom	GCAAAAAAAATTGAGATAAAATTACAATAAAATTATAAAC	
40 bp S18 (72.5% GC)		DNA binding
Top	/5FluorT/ TCCAGACCAGAGGAGCTCCAGCGCTGGGAGCGGCCAGGGC	
Bottom	GCCCTGGCCGCTCCCAGCGCTGGAGCTCCTCTGGTCTGGA	
60 bp AT-rich (31.7% GC)		cryo-EM<br>ATPase<br>pull-down
Top	CCTGCAGGCCTTTTGAAAAGCAAGCATAAAAGATCTAAACATAAAATCTGTAAAATAACA	
Bottom	TGTTATTTTACAGATTTTATGTTTAGATCTTTTATGCTTGCTTTTCAAAAGGCCTGCAGG	
60 bp GC-rich (70% GC)		cryo-EM
Top	GGGGCTCGACTCCAAGTGCGGGGCACTCGTCTGTGAGGGGGCTCAGTTCGCACTGCGGGG	
Bottom	CCCCGCAGTGCGAACTGAGCCCCCTCACAGACGAGTGCCCCGCACTTGGAGTCGAGCCCC	
60 bp poly(dA·dT)		cryo-EM
Top	AAAAAAAAAAAAAAAAAAAAAAAAAAAAAAAAAAAAAAAAAAAAAAAAAAAAAAAAAAAA	
Bottom	TTTTTTTTTTTTTTTTTTTTTTTTTTTTTTTTTTTTTTTTTTTTTTTTTTTTTTTTTTTT	

84 bp AT-rich (32.1% GC)		
Top	TTTGTGCACTTGCCTGCAGGCCTTTTGAAAAGCAAGCATAAAAGATCTAAACATAAAATCTGT<br>AAAATAACAAGATGTAAAGAT	cryo-EM
Bottom	ATCTTTACATCTTGTTATTTTACAGATTTTATGTTTAGATCTTTTATGCTTGCTTTTCAAAAGGC<br>CTGCAGGCAAGTGCACAAA	
178 bp AT-rich (30.9% GC)		
Top	/5Biosg/GCCGGCATTTAAATCAAAATAGCAAATTTCGTCAAAAATGCTAAGAAATAGGTTATTA<br>CTGAGTAGTATTTATTTAAGTATTGTTTGTGCACTTGCCTGCAGGCCTTTTGAAAAGCAAGCA<br>TAAAAGATCTAAACATAAAATCTGTAAAATAACAAGATGTAAAATTTAAATCGCCGG	Mcm2-7 loading
Bottom	/5Biosg/CCGGCGATTTAAATTTTACATCTTGTTATTTTACAGATTTTATGTTTAGATCTTTTATG<br>CTTGCTTTTCAAAAGGCCTGCAGGCAAGTGCACAAACAATACTTAAATAAATACTACTCAGTA<br>ATAACCTATTTCTTAGCATTTTTGACGAAATTTGCTATTTTGATTTAAATGCCGGC	
178 bp poly(dA·dT)		
Top	/5Biosg/GCCGGCATTTAAATAAAAAAAAAAAAAAAAAAAAAAAAAAAAAAAAAAAAAAAAAAAA<br>AAAAAAAAAAAAAAAAAAAAAAAAAAAAAAAAAAAAAAAAAAAAAAAAAAAAAAAAAAAAAAAA<br>AAAAAAAAAAAAAAAAAAAAAAAAAAAAAAAAAAAAAAAAAAATTTAAATCGCCGG	Mcm2-7 loading
Bottom	/5Biosg/GCCGGCATTTAAATTTTTTTTTTTTTTTTTTTTTTTTTTTTTTTTTTTTTTTTTTTTTTTTT<br>TTTTTTTTTTTTTTTTTTTTTTTTTTTTTTTTTTTTTTTTTTTTTTTTTTTTTTTTTTTTTTTTTTTTTT<br>TTTTTTTTTTTTTTTTTTTTTTTTTTTTTTTATTTAAATCGCCGG	

5FluorT – 5’ Fluorescein dT label; 5Biosg – 5’ Biotin label

Supplementary References

1. Bleichert, F., Botchan, M.R. & Berger, J.M. Crystal structure of the eukaryotic origin recognition complex. *Nature* **519**, 321-6 (2015).

2. Bleichert, F., Leitner, A., Aebersold, R., Botchan, M.R. & Berger, J.M. Conformational control and DNA-binding mechanism of the metazoan origin recognition complex. *Proc Natl Acad Sci U S A* **115**, E5906-E5915 (2018).

3. Ranjan, A. & Gossen, M. A structural role for ATP in the formation and stability of the human origin recognition complex. *Proc Natl Acad Sci U S A* **103**, 4864-9 (2006).

4. Tocilj, A. et al. Structure of the active form of human origin recognition complex and its ATPase motor module. *eLife* **6**, e20818 (2017).

5. Siddiqui, K. & Stillman, B. ATP-dependent assembly of the human origin recognition complex. *J Biol Chem* **282**, 32370-83 (2007).

6. Li, N. et al. Structure of the origin recognition complex bound to DNA replication origin. *Nature* **559**, 217-222 (2018).
