# Supplementary material for: Structural mechanism for replication origin binding and remodeling by a metazoan origin recognition complex and its co-loader Cdc6
Source: Nat Commun. 2020 Aug 26;11:4263. doi: 10.1038/s41467-020-18067-7 (PMC7450096; doi:10.1038/s41467-020-18067-7)
Supplement: Supplementary file 4 — Description of Additional Supplementary Files [file 41467_2020_18067_MOESM4_ESM.pdf]

Description of Additional Supplementary Files

File name: Supplementary Movie 1

Description: Structure overview of *Drosophila* ORC bound to DNA and Cdc6. The D*m*ORC·DNA·Cdc6 model (60 bp AT-rich DNA) is shown and DNA binding elements in the AAA+ and WH domains are highlighted. DNA regions in *Drosophila* ORC are also compared to those in *S. cerevisiae* ORC (PDB 5zr1[20]).
