# Supplementary material for: Structural mechanism for replication origin binding and remodeling by a metazoan origin recognition complex and its co-loader Cdc6
Source: Nat Commun. 2020 Aug 26;11:4263. doi: 10.1038/s41467-020-18067-7 (PMC7450096; doi:10.1038/s41467-020-18067-7)
Supplement: Supplementary file 2 — Peer Review File [file 41467_2020_18067_MOESM2_ESM.pdf]

REVIEWERS' COMMENTS:

Reviewer #1 (Remarks to the Author):

JM Schmidt and F Bleichert report a structural mechanism for replication origin binding and remodeling by the Drosophyla melanogaster's Origin Recognition Complex (ORC) and its co-loader Cdc6. While ORC recognizes consensus DNA sequences in S. cerevisiae, metazoan ORC (such as D. melanogaster) associates with chromosomal DNA independently of specific sequence elements. This work aims to decipher the structural basis for DNA engagement by ORC in the absence of specific consensus DNA sequences.
The authors report near atomic resolution cryo-EM structures of D. melanogaster ORC bound to different DNA substrates in the absence and presence of its co-loader Cdc6. These structures show how the ATPase modules of subunits Orc1 and Orc4 constitute the primary DNA binding site in the center of the ORC ring and cooperate with the winged-helix domains to stabilize DNA bending. Correlating structural observations with biochemical data further shows that DNA sequence modulates DNA binding and remodeling, and that DNA bending in turn promotes Mcm2-7 loading. To explain the distinct DNA sequence-dependencies of S. cerevisiae and D. melanogaster (metazoan), the DNA contacts between S. cerevisiae and metazoan ORC are extensively compared. These findings support a model in which DNA geometry and DNA bendability contribute to the selection of chromosomal Mcm2-7 helicase loading sites in metazoans.
This work answers fundamental questions about the initiation of DNA replication in eukaryotes. The manuscript is well-written, the figures are nice and informative. The conclusions are sound and based on an impressive amount of functional and structural data. Please find below a few points that the authors should consider when preparing the revised version of their manuscript.

Major points:

The DNA contacts are extensively described. My main concern is that non-specialist readers may find it difficult to capture some of the article's important conclusions.
- Figure 7 summaries the main conclusions of the article. The figure is very nice and informative. However, the figure may be extended by detailing and numbering the different conformational transitions in metazoan ORC and DNA remodeling.
- A movie may be useful to compare the DNA contacts between S. cerevisiae and metazoan ORC, and explain the distinct DNA sequence-dependencies of S. cerevisiae and D. melanogaster.

Minor points:
- P.2: … the eukaryotic kingdom… should be … the eukaryotic domain of life … (The taxonomic rank of Eukarya is a domain not a kingdom).
- P.9: "backbone phosphates" may be "phosphate backbones".
- P.11: You may consider splitting the following sentence: "Two notable exceptions include the WH domain of Orc2, which is detached from the AAA+ layer and flexible in the absence of Cdc6 (Extended Data Figures 6E and 6G), and the arginine finger helix in Orc1, a region that interacts with the Cdc6 AAA+ module in the ternary complex and is less ordered in the DNA-free DmORC and the DmORC·DNA assembly, suggesting it undergoes a disorder-to-order transition upon Cdc6 binding."
- "van der Waals contacts" or "van der Waals interactions" is used in many occasions in the manuscript to describe the interactions within the different protein complexes. Hydrophobic interactions may be more appropriate ?
- The interactions within the different protein complexes are extensively described. However several sentences may be confusing for the readers. The following sentences may be revised:
P.7: In agreement with this evolutionary kinship, Orc1 and Cdc6 are predicated on the same protein folds and adopt similar 3D structures in the ternary initiator·co-loader complex.
P.9: The ISMs of Orc1, Orc4, and Cdc6 in particular form several hydrogen bonds and van der Waals contacts with the sugar-phosphate backbone of one of the DNA strands that together extend for half a

turn of the duplex
- P31-32 (mat & meth): imidazole p31 … or … Imidazole p32 : please homogenize.

Reviewer #2 (Remarks to the Author):

Schmidt and Bleichert report the reconstitution of the MCM helicase loading reaction in vitro, using purified Drosophila proteins, and the cryo-EM structure of the Drosophila ORC-DNA and ORC-DNA-Cdc6 complex. They find that, in both complexes, DNA is bent as it traverses the central pore of the ORC ring. The ORC-Cdc6 ring forms a flat ring configuration, structurally distinct from previous helicase loading intermediates observed in yeast. The ORC-Cdc6-DNA complex also contains a conformation distinct from the auto-inhibited state previously observed for the isolated ORC complex. Using structural and biochemical tools, the authors describe a new B-loop element in Orc1 that is responsible for the high-affinity interaction of Drosophila ORC with DNA. The authors also describe the cryo-EM structures of DmORC-Cdc6 bound to DNA of different sequence compositions. Interestingly, they establish, that duplex DNA segments differing in sequence can be bent to different degrees, and that DNA bendability affects MCM loading efficiency.

This work is simply outstanding. The in vitro reconstitution of the MCM loading with metazoan proteins alone would grant in my opinion publication in a high impact journal. In addition to this however, the authors report an extremely high quality structural and biochemical study, describing important physical aspects of helicase loading by ORC.

I only have a few minor comments:

1. The manuscript in general is very clearly written, although I must say in certain passages the language appears unnecessarily complicated. For example, in the abstract, "a nucleotidyl hydrolase that uses adenosine triphosphate (ATP) binding and turnover to control its association with chromosomal regions" could be summarized with "a DNA binding ATPase". I would suggest that the authors critically read their manuscript to eliminate such passages, to improve an otherwise exquisitely written manuscript.

2. I would also recommend that the authors put a bigger emphasis on their achievement of the reconstitution of MCM loading using purified Drosophila proteins. Again, this is probably the most significant aspect of the work presented, and at the moment it feels like something appended to the study, rather than its central piece.

3. Negative stain EM micrographs should be added as supplementary material for the MCM loading.

4. The authors describe an ORC-Cdc6 complex that exists in a flat configuration that is different from the spiral form described for the yeast OCCM, and they speculate that MCM engagement could reconfigure the ORC-Cdc6. Could the authors clarify to what extent the ATPase sites in the Orc-Cdc6 complex would be altered?

Response to reviewers:

We thank the referees for their time to carefully review our manuscript and their insightful comments. We are grateful for the positive sentiments evinced by both reviewers and their strong enthusiasm for our work.

In the enclosed revision, we have addressed all concerns and suggestions of both referees. A detailed description of these changes and a point-by-point response to all issues raised is detailed in blue below.

Reviewer #1:

JM Schmidt and F Bleichert report a structural mechanism for replication origin binding and remodeling by the Drosophyla melanogaster's Origin Recognition Complex (ORC) and its co-loader Cdc6. While ORC recognizes consensus DNA sequences in S. cerevisiae, metazoan ORC (such as D. melanogaster) associates with chromosomal DNA independently of specific sequence elements. This work aims to decipher the structural basis for DNA engagement by ORC in the absence of specific consensus DNA sequences.

The authors report near atomic resolution cryo-EM structures of D. melanogaster ORC bound to different DNA substrates in the absence and presence of its co-loader Cdc6. These structures show how the ATPase modules of subunits Orc1 and Orc4 constitute the primary DNA binding site in the center of the ORC ring and cooperate with the winged-helix domains to stabilize DNA bending. Correlating structural observations with biochemical data further shows that DNA sequence modulates DNA binding and remodeling, and that DNA bending in turn promotes Mcm2-7 loading. To explain the distinct DNA sequence-dependencies of S. cerevisiae and D. melanogaster (metazoan), the DNA contacts between S. cerevisiae and metazoan ORC are extensively compared. These findings support a model in which DNA geometry and DNA bendability contribute to the selection of chromosomal Mcm2-7 helicase loading sites in metazoans.

This work answers fundamental questions about the initiation of DNA replication in eukaryotes. The manuscript is well-written, the figures are nice and informative. The conclusions are sound and based on an impressive amount of functional and structural data. Please find below a few points that the authors should consider when preparing the revised version of their manuscript.

Major points:

-The DNA contacts are extensively described. My main concern is that non-specialist readers may find it difficult to capture some of the article's important conclusions.

We thank the reviewer for this comment. We have substantially shortened the description of the DNA contacts as well as the structural comparison between ORC·DNA·Cdc6, ORC·DNA, and DNA-free ORC structures. We hope that condensing these sections will help readers capture the most important findings.

- Figure 7 summaries the main conclusions of the article. The figure is very nice and informative. However, the figure may be extended by detailing and numbering the different conformational transitions in metazoan ORC and DNA remodeling.

We thank the reviewer for this suggestion. We have added numbers to individual steps in the scheme in **Fig. 7a** and refer to them at the relevant places in the discussion. We hope this makes it easier for readers to spot the conformational transitions described.

- A movie may be useful to compare the DNA contacts between S. cerevisiae and metazoan ORC, and explain the distinct DNA sequence-dependencies of S. cerevisiae and D. melanogaster.

We have included a movie with the revision as suggested.

Minor points:

- P.2: … the eukaryotic kingdom… should be … the eukaryotic domain of life … (The taxonomic rank of Eukarya is a domain not a kingdom).

We have replaced ‘kingdom’ with ‘domain’.

- P.9: “backbone phosphates” may be “phosphate backbones”.

We have modified the wording in this sentence.

- P.11: You may consider splitting the following sentence: “Two notable exceptions include the WH domain of Orc2, which is detached from the AAA+ layer and flexible in the absence of Cdc6 (Extended Data Figures 6E and 6G), and the arginine finger helix in Orc1, a region that interacts with the Cdc6 AAA+ module in the ternary complex and is less ordered in the DNA-free DmORC and the DmORC·DNA assembly, suggesting it undergoes a disorder-to-order transition upon Cdc6 binding.”

To comply with space limitations, we have substantially shortened this section of the manuscript, which included removing this sentence.

- “van der Waals contacts” or “van der Waals interactions” is used in many occasions in the manuscript to describe the interactions within the different protein complexes. Hydrophobic interactions may be more appropriate?

We do not observe any hydrophobic interactions contributing to ORC/DNA contacts in the reported structures. However, we have removed some of the details when describing ORC-DNA interactions, which also reduces the frequency of us using these terms.

- The interactions within the different protein complexes are extensively described. However several sentences may be confusing for the readers. The following sentences may be revised: P.7: In agreement with this evolutionary kinship, Orc1 and Cdc6 are predicated on the same protein folds and adopt similar 3D structures in the ternary initiator·co-loader complex.

P.9: The ISMs of Orc1, Orc4, and Cdc6 in particular form several hydrogen bonds and van der Waals contacts with the sugar-phosphate backbone of one of the DNA strands that together extend for half a turn of the duplex

We have revised both sentences and hope that they are less confusing now.

- P31-32 (mat & meth): imidazole p31 … or … Imidazole p32 : please homogenize.

We have corrected these typos.

Reviewer #2:

Schmidt and Bleichert report the reconstitution of the MCM helicase loading reaction in vitro, using purified Drosophila proteins, and the cryo-EM structure of the Drosophila ORC-DNA and ORC-DNA-Cdc6 complex. They find that, in both complexes, DNA is bent as it traverses the central pore of the ORC ring. The ORC-Cdc6 ring forms a flat ring configuration, structurally distinct from previous helicase loading intermediates observed in yeast. The ORC-Cdc6-DNA complex also contains a conformation distinct from the auto-inhibited state previously observed for the isolated ORC complex. Using structural and biochemical tools, the authors describe a new B-loop element in Orc1 that is responsible for the high-affinity interaction of Drosophila ORC with DNA. The authors also describe the cryo-EM structures of DmORC-Cdc6 bound to DNA of different sequence compositions. Interestingly, they establish, that duplex DNA segments differing in sequence can be bent to different degrees, and that DNA bendability affects MCM loading efficiency.

This work is simply outstanding. The in vitro reconstitution of the MCM loading with metazoan proteins alone would grant in my opinion publication in a high impact journal. In addition to this however, the authors report an extremely high quality structural and biochemical study, describing important physical aspects of helicase loading by ORC.

I only have a few minor comments:

1. The manuscript in general is very clearly written, although I must say in certain passages the language appears unnecessarily complicated. For example, in the abstract, "a nucleotidyl hydrolase that uses adenosine triphosphate (ATP) binding and turnover to control its association with chromosomal regions" could be summarized with "a DNA binding ATPase". I would suggest that the authors critically read their manuscript to eliminate such passages, to improve an otherwise exquisitely written manuscript.

We thank the reviewer for this comment. We have carefully read our manuscript and shortened several passages.

2. I would also recommend that the authors put a bigger emphasis on their achievement of the reconstitution of MCM loading using purified Drosophila proteins. Again, this is probably the most significant aspect of the work presented, and at the moment it feels like something appended to the study, rather than its central piece.

We appreciate the reviewer's enthusiasm for our *in vitro* reconstitution of *Drosophila* Mcm2-7 loading. We have added references to these experiments in the abstract, introduction, and discussion. We have also shortened other sections of the main text, which we hope will help put more emphasis on Mcm2-7 loading.

3. Negative stain EM micrographs should be added as supplementary material for the MCM loading.

We have included a negative stain micrograph of loaded Mcm2-7 as a new panel in **Supplemental Fig. 1e**.

4. The authors describe an ORC-Cdc6 complex that exists in a flat configuration that is different from the spiral form described for the yeast OCCM, and they speculate that MCM engagement could reconfigure the ORC-Cdc6. Could the authors clarify to what extent the ATPase sites in the Orc-Cdc6 complex would be altered?

The reviewer raises an interesting point. Globally, the ATPase sites are similar. However, due to the lower resolution of the OCCM structure and the weak density for Cdc6 in the OCCM, the positions of most of the amino acid side chains remain ambiguous, preventing a detailed comparison. As we are also limited for space, we have decided to remove this sentence from the revised manuscript.
